# Fluorescence-Based Sensing of Pesticides Using Supramolecular Chemistry

**DOI:** 10.3389/fchem.2021.616815

**Published:** 2021-04-16

**Authors:** Mindy Levine

**Affiliations:** Ariel University, Department of Chemical Sciences, Ariel, Israel

**Keywords:** pesticides, fluorescence spectroscopy, supramolecular chemistry, macrocycles, polymers, nanoparticles

## Abstract

The detection of pesticides in real-world environments is a high priority for a broad range of applications, including in areas of public health, environmental remediation, and agricultural sustainability. While many methods for pesticide detection currently exist, the use of supramolecular fluorescence-based methods has significant practical advantages. Herein, we will review the use of fluorescence-based pesticide detection methods, with a particular focus on supramolecular chemistry-based methods. Illustrative examples that show how such methods have achieved success in real-world environments are also included, as are areas highlighted for future research and development.

## Introduction

Worldwide, pesticides, including insecticides, fungicides, herbicides, rodenticides, bactericides, and larvicides, enjoy widespread usage and high levels of commercial success (Yousefi et al., 2020). Such commercial success stems from the fact that pesticides have been instrumental in reducing the destruction of crops (Damalas, 2009), and are responsible for increasing world-wide food production (Richter and Safi, 1997) and mitigating the problem of food insufficiencies, including among children and other vulnerable populations (Bonner and Alavanja, 2017). Moreover, pesticide usage continues to increase: Whereas in 1999, approximately 5.6 billion pounds of pesticides were used worldwide annually by 1.8 billion people (Donaldson et al., 1998), these numbers are expected to increase to 3.5 million tons (7 billion pounds) by the end of the year 2020 (Sharma et al., 2019).

Despite the significant benefits associated with the use of pesticides, they have a number of concurrent deleterious health effects. In one historical example, agricultural researchers championed the use of the pesticide dichlorodiphenyltrichloroethane, a synthetic insecticide that was produced in the 1940s, in public awareness campaigns that claimed that, “DDT is good for me!” (Conis, 2017). The initial positive publicity related to the efficacy of DDT as an insecticide was soon mitigated by the publication of “Silent Spring” by Rachel Carson in 1962, which highlighted numerous deleterious health effects of DDT (Parks, 2017), resulting in increasing regulation around DDT’s usage (Morris, 2019). Because DDT is extraordinarily environmentally persistent, however, measurable quantities of DDT are still being detected in the environment, even after the United States’ and worldwide bans of DDT were enacted (Booij et al., 2016). Moreover, although the most common isomer of DDT (*para, para*) has been banned, other isomers are still in use; the toxicity of these isomers is largely unknown (Niu et al., 2018). Finally, overall regulation surrounding the use of pesticides varies significantly worldwide, with the global ban on DDT enacted by the Stockholm convention in 2001. Other pesticides face broad variability, both in terms of the legislation around their usage and in the maximum regulatory limits (MRLs) permitted for the pesticides in food and other commercial products (Pang et al., 2015).

Subsequent generations of pesticides were designed to maintain high efficacy in combating pests that could destroy crops, while reducing the toxicity of the pesticides to humans, animals and the environment. These efforts have had mixed success, with negative health effects associated with exposure to organophosphorus-containing pesticides (Eddleston et al., 2008), triazole-derived pesticides (Filipov and Lawrence, 2001), carbamate-containing pesticides (Haendel et al., 2004), and nearly every generation of pesticides commercialized to date (Fierascu et al., 2020; Oka, 2020). Partially as a result of the toxicity of virtually every commercially available pesticide, consumers began gravitating towards the organic farming industry, which was purported to use less toxic exogenous pesticides to obtain analogous agricultural results (Raj et al., 2019). Nonetheless, the materials used by organic farmers have nontrivial toxicity, and the high concentrations of those materials that are needed in order to achieve efficacy lead to negative health effects (van Bruggen and Finckh, 2016; Mossa et al., 2018). Moreover, even minimal toxicity in pesticides is compounded by their long environmental persistence (Bilala et al., 2019), which means that a single application of pesticides can result in exposure to pesticide residue years later.

As a result of the toxicity of the pesticides, their ubiquitous usage, and their environmental persistence, there exists an ongoing and pressing public health need to develop detection methods for pesticides that are: 1) **sensitive** for low concentrations of pesticides; 2) **selective** for detecting the pesticides, even in the presence of structurally similar species that are present in higher concentrations; 3) **robust** and **generally applicable** for detecting pesticides in complex, real-world environments; and 4) **practical** for usage by untrained professionals with a low cost to detection and a rapid signal response.

Currently used detection methods for these pesticides overwhelmingly rely on the use of mass spectrometry-based techniques (Fu et al., 2019), including gas chromatography-mass spectrometry (GC-MS) (Mondal et al., 2018), liquid chromatography-mass spectrometry (LC-MS) (Stachniuk et al., 2017), and inductively coupled plasma-mass spectrometry (ICP-MS) (Gui et al., 2016). While these methods have been successful in providing unparalleled sensitivity to detect minute quantities of pesticides (Moschet et al., 2014), they also have significant disadvantages. These notable disadvantages include challenges in implementing such methods in on-site pesticide detection efforts (Lopez et al., 2014), as well as the need for costly laboratory instrumentation and for trained technicians to operate such instrumentation and interpret the results (Bush, 2019). Other disadvantages to traditional detection methods include: 1) challenges of selectivity, wherein common co-existing but benign analytes can provide false positive signals using the pesticide sensor (Bexfield et al., 2020); 2) challenges in applicability for a pesticide life cycle, in which the sensor can detect the pesticide but not its decomposition products (Chen et al., 2019b); and 3) challenges in obtaining rapid results, due to the need for time-consuming separation and purification procedures, which are particularly critical in rapidly changing, real-world environments. Of note, many of these challenges can be addressed using on-site sampling followed by detailed in-laboratory analysis; such a strategy breaks down, however, for highly unstable pesticide and pesticide-related analytes (Valenzuela et al., 2020).

Moreover, an overreliance on the use of mass spectrometry-based techniques can lead to critical knowledge gaps in real-world detection. Such challenges, in turn, have led to the development of other methods for pesticide detection, including those that operate based on changes in Raman spectra, electrochemical signals (Arduini et al., 2019), UV-visible absorbance spectra (Li et al., 2018a), and fluorescence spectra (Nsibande and Forbes, 2016). Among these novel pesticide detection systems, fluorescence-based sensing has significant advantages, based on the known ability of fluorescence-based sensors to provide high sensitivity, robust signal-to-noise ratios, and rapid response times, even in real-world environments (Chen et al., 2018a; Bigdeli et al., 2019). Indeed, the use of fluorescence-based sensors for pesticide detection has benefited from substantial research attention in recent years (Yan et al., 2018a), as researchers understand how such sensors can benefit ongoing pesticide detection efforts.

Reviewed herein is precisely this research area: fluorescence-based sensors for pesticides, with a particular focus on supramolecular sensors. We start by briefly reviewing categories of pesticides, focusing on structural differences between the pesticides that will necessarily inform sensing strategies (*Review of Pesticide Categories*). From there we will move to a discussion of fluorescence-based detection mechanisms (*Review of Fluorescence Detection Mechanisms*), including fluorescence quenching, fluorescence enhancement, and various forms of fluorescence energy transfer. The main part of this review article, Different scaffolds for pesticide detection, deals with supramolecular scaffolds used for fluorescence-based detection of pesticides, including the use of fluorescent polymers (*Fluorescent Polymer-Based Pesticide Detection*), macrocycles (*Macrocycle-Based Pesticide Detection*), nanomaterials (*Nanomaterial-Based Pesticide Detection*), and metal-organic frameworks (*Metal-Organic Framework Pesticide Detection*). Importantly, this review does not claim to provide a comprehensive review of all supramolecular scaffolds used for pesticide detection; for example, scaffolds such as DNA-containing aptamers have been used effectively for pesticide detection but are not discussed explicitly herein (Jiang et al., 2020b) Because fluorescence-based pesticide detection strongly depends on the physical phase in which the pesticide is being detected (vapor, liquid, or solid), Pesticide detection in different phases deals explicitly with how phase differences inform and require differences in detection strategy. Finally, the conclusion (*Conclusion and Future Outlook*) discusses ongoing unsolved pesticide detection challenges.

There are a number of other excellent reviews in the area of fluorescence-based pesticide detection, including those that focus on nanoparticles (Singh et al., 2020), polymers (Farooq et al., 2018), and metal-containing scaffolds (Mishra et al., 2018), as well as reviews in the general area of fluorescence-based chemosensors (Mako et al., 2019) and other mechanisms of pesticide detection (Llorent-Martínez et al., 2011; Van Dyk and Pletschke, 2011; Verma and Bhardwaj, 2015). Interested readers are directed towards these reviews for more in-depth analysis of these particular research areas. The author also wishes to apologize to those researchers whose work was inadvertently overlooked in the preparation of this manuscript.

### Review of Pesticide Categories

**FIGURE 1:**
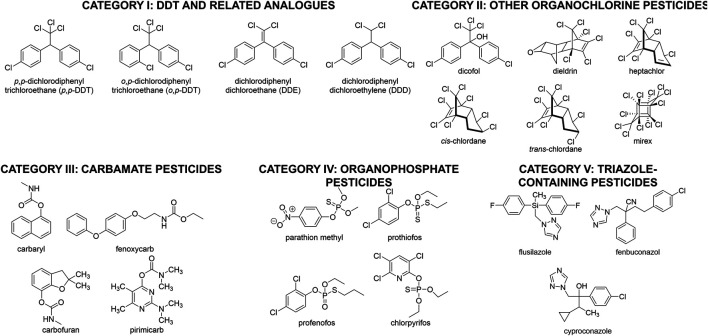
Different structural classes of pesticides that will be discussed herein.

There are a broad variety of pesticides that are currently in use, and even more that have been used historically but are no longer in use. These pesticides can be characterized by chemical structure, which assists in the design of structure-based pesticide sensors. Historically, chemicals such as sulfur (Hagstrum and Phillips, 2017), arsenic (Hughes et al., 2011), and lead (Lu et al., 2020a) were used to control pests, although reports indicate that unintentional human toxicity was widespread (Raanan et al., 2017). By the 20th century, the agricultural industry was predominantly using DDT and DDT analogues (Figure 1, Category I) (Maldonado et al., 2012). These highly chlorinated, diphenylmethane structures were successful in mitigating the threat from a broad variety of pests (Kasinathan et al., 2019). Nonetheless, reports of deleterious health effects of DDT and DDT analogues to humans (Zeinomar et al., 2020), animals, and the environment (Kabasenche and Skinner, 2014), including concerns that DDT could be carcinogenic (Deziel et al., 2021), led to its regulatory ban in the United States in 1972 (Wang et al., 2020c). Of note, despite a worldwide ban on agricultural usage issued by the 2001 Stockholm Convention, certain countries still use DDT in small quantities and under tightly regulated conditions (Mishra and Sharma, 2011).

Replacements for the use of DDT focused on a variety of other halogenated small molecules (Figure 1, Category II), including dicofol (Yin et al., 2017), dieldrin, heptachlor, chlordane, and mirex (Varnosfaderany et al., 2020). Although these compounds maintained high efficacy against a variety of pests, they were nonetheless abandoned due to their high toxicity (Martyniuk et al., 2020), including suspected genotoxic damage to children (Anguiano-Vega et al., 2020), and suspected carcinogenic effects (Suarez-Torres et al., 2020). In an attempt to avoid unwanted toxicity, pesticide researchers next developed several classes of non-halogenated pesticide structures, including carbamates (Figure 1, Category III) (Kappaun et al., 2018), phosphates (Figure 1, Category IV) (Gonzalez-Alzaga et al., 2020), and triazoles (Figure 1, Category V) (Bele and Singhvi, 2011). While researchers had some success with these insecticides, nonetheless the suspected toxicity of these structures (particularly the phosphates) led to concerns about their long-term usage as well (Tutuncu et al., 2019; Meenu et al., 2020). Moreover, nearly all pesticides investigated have significant long-term environmental persistence, which means that their environmental impact can last for decades even after their usage is discontinued (Wang et al., 2019a; Kaushal et al., 2021).

Overall, despite significant efforts to develop nontoxic yet still effective pesticides, researchers have struggled. Partially as a result of such struggles, attempts to control pests *via* “organic” methods have gained in popularity in recent years, and include the use of copper materials (Gazzurelli et al., 2020), boric acid (Sierras et al., 2018), and hydrogen peroxide (Bajwa and Sandhu, 2014). While there is some evidence that these materials may be less toxic to both humans and the environment, the high concentrations of organic pesticides necessary to achieve efficacy raise questions about the overall benefit of their usage (Tsatsakis et al., 2016). Current best agricultural practices tend towards combining pesticide usage with practices such as integrated pest management and crop rotation (MacIntosh, 2019; Singh et al., 2019; Smith and Chalk, 2020) to limit exogenous pesticide use.

### Review of Fluorescence Detection Mechanisms

**FIGURE 2:**
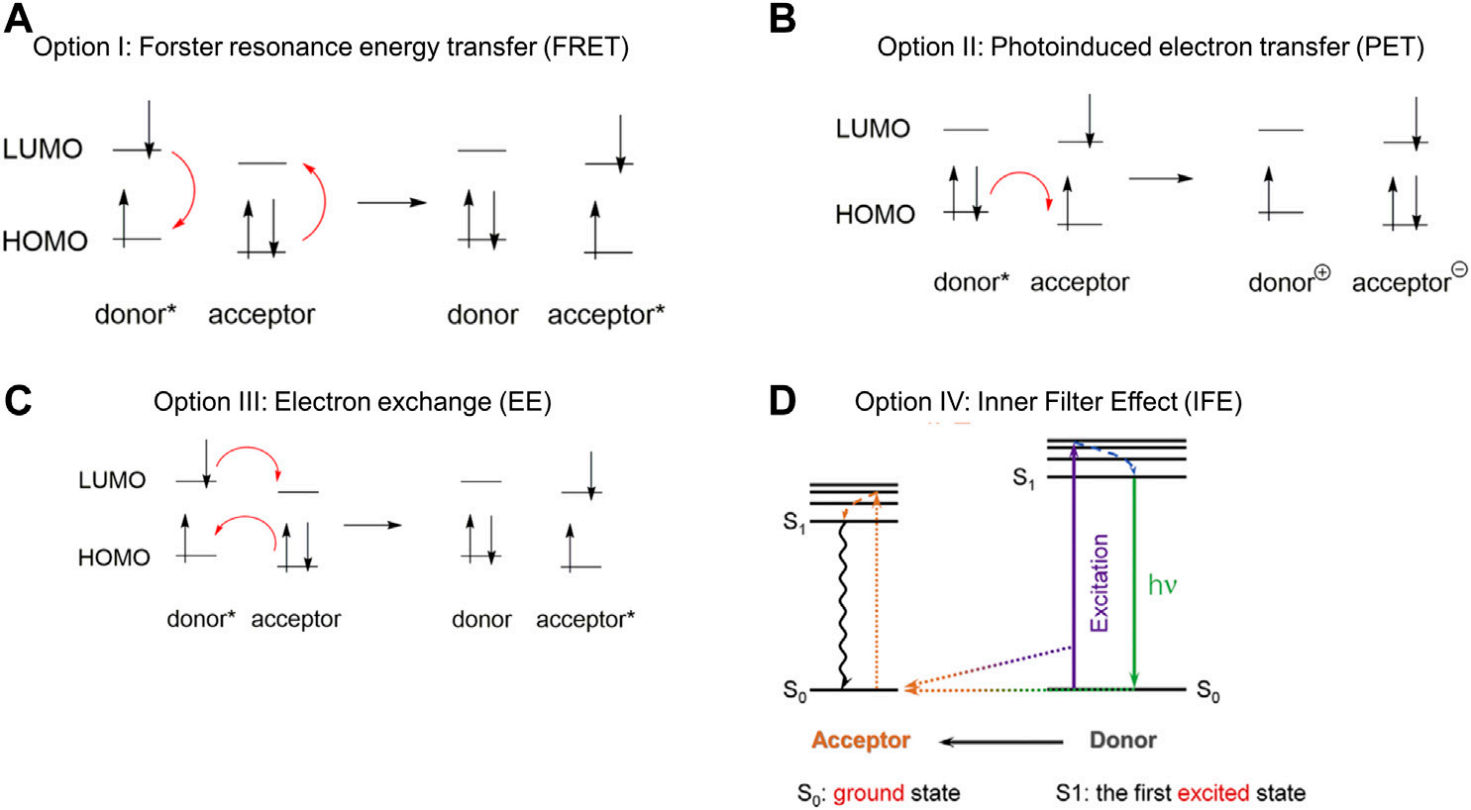
Molecular orbital diagrams representing **(A)** Forster resonance energy transfer from an excited state donor to an acceptor **(B)** Photoinduced electron transfer; and **(C)** Electron exchange **(D)** Schematic illustration of the inner filter effect. Reprinted from references Mako et al. (2019) and Zhang et al. (2019a).

Fluorescence detection can occur through a variety of mechanisms, with changes in the fluorescence signal of the system acting as the transducing element. A variety of mechanisms of fluorescence detection have been reviewed in other publications (Bissell et al., 2005; Kumar Panigrahi and Kumar Mishra, 2019; Cao et al., 2020; Wu et al., 2020), and the interested reader is directed towards these references for more information. In general, changes in the fluorescence signal upon introduction of pesticides are most often “turn-off” changes, resulting from pesticide-induced fluorescence quenching. Such quenching occurs through energy transfer from the sensor to the pesticide, which is a result of Förster resonance energy transfer (FRET, Figure 2, Option I); photoinduced electron transfer (PET, Figure 2, Option II); electron exchange (EE, Figure 2, Option III); or an inner filter effect (IFE, Figure 2, Option IV). Each of these mechanisms is discussed briefly below.

Option 1: Förster resonance energy transfer (FRET). Förster resonance energy transfer theory treats the energy donor and acceptor as two dipoles that interact at relatively long distances (Saini et al., 2009). A main requirement of FRET is the need for spectral overlap between the emission spectrum of the energy donor and the absorption spectrum of the acceptor, quantified as the spectral overlap integral, *J* (Talukdar and Kundu, 2019). While this mechanism of energy transfer has been used extensively, practical challenges in using FRET for fluorescence sensing relate to the need for spectral overlap and the fact that there is often undesired overlap between the emission spectra of the donor and the acceptor. Such unintended overlap can compromise the sensitivity of fluorescence-based turn-on sensors, as even in the absence of energy transfer residual donor emission may be observed (Hsu et al., 2017). In general, FRET has been used as an effective energy transfer mechanism that enables pesticide detection, mostly in cases in which the pesticide acts as the FRET acceptor and causes a fluorescence quenching upon effective energy transfer (Chen et al., 2020c).

Option 2: Photoinduced electron transfer (PET). PET refers to cases in which an electron is transferred directly from the energy donor to the energy acceptor, as opposed to FRET in which no electron is transferred (Rafiq and Scholes, 2019). Such a transfer is initiated by photochemical excitation, and requires reasonably close proximity between the donor and acceptor (Kim et al., 2020). PET can occur both through bonds (Zhang et al., 2020c) and through space (Gao et al., 2020), and fluorescence sensing using PET-type mechanisms has been extensively reported (Wei et al., 2020b; Yuvaraj et al., 2020). Pesticide detection that occurs through PET generally operates through a mechanism in which the introduction of the pesticide disrupts PET that is already occurring, leading to a signal enhancement, signal quenching, or signal change, depending on the nature of the donor and acceptor involved in the PET mechanism (Zhang et al., 2020b).

Option 3: Electron exchange (EE). EE is also referred to as Dexter energy transfer, and refers to situations in which an electron is transferred from the LUMO of an excited state donor to the LUMO of an acceptor at the same time that an electron is transferred from the HOMO of the acceptor to the HOMO of the donor (Liao et al., 1995). EE requires orbital overlap and concomitant close contact between the donor and the acceptor (Likhtenshtein, 1996), and has been implicated in a variety of energy transfer schemes. EE is particularly effective in situations in which the donor and acceptor can be trapped in conformations that facilitate the necessary close contact (Campanelli and Domenicano, 2019), such as when supramolecular binding inside macrocycle enforces the close-range interactions (Soler and McCusker, 2008). Compared to FRET, EE is markedly less dependent on the spectral overlap integral J, and therefore has the potential to lead to turn-on fluorescence detection with a completely dark background (i.e. without residual donor emission) (Zheng and Swager, 2004). Pesticides are generally highly electron deficient, which means that they act as electron acceptors in EE mechanisms, although the radicals that result from the pesticide’s acceptance of a single electron are generally detected using mass spectrometry-based techniques (Edgell et al., 1993).

Option 4: Inner filter effect (IFE). The IFE refers to situations in which, like with FRET, there is significant spectral overlap between the donor and acceptor (Zhang et al., 2019). In this case, however, the energy acceptor absorbs excited state energy and attenuates how much of that energy can reach the luminescent species. While such an effect has historically been seen as undesirable in that in limits emission from the target species (Thompson and Scarlata, 2019), control over the IFE based on rational system design has enabled it to be used for effective fluorescence-based sensing (Marks et al., 2015). IFE has achieved significant success in facilitating pesticide detection, mostly due to the high absorbance of the pesticide in the UV spectral region that provides an internal, (i.e. inner) filter of the light that reaches the target luminophore (Sharma et al., 2020).

By far the most common sensor response to the introduction of a pesticide is to respond with a decrease in the observed fluorescence emission. Nonetheless, certain options for pesticide-induced fluorescence enhancements remain. For example, pesticide-induced fluorescence increases can occur in cases where the pesticide causes aggregation, for example, with sensors that participate in aggregation induced emission (AIE) (Han et al., 2020b). While aggregation more commonly leads to decreased fluorescence emission due to the formation of non-fluorescent aggregates (Zhang et al., 2019b), particular sensor structures enable aggregation induced emission enhancements (Tarai et al., 2020).

Finally, many pesticides are weakly fluorescent, usually with quantum yields that preclude direct observation of their fluorescence emission (Da Silva et al., 2001). Binding of the pesticide to a sensor, particularly macrocyclic sensors, results in rigidification of the local environment and a decrease in the availability of non-radiative decay pathways (Cucolea et al., 2015). As a result, the pesticide’s quantum yield increases and it can be detected through direct monitoring of its (now enhanced) emission signal.

## Different Scaffolds for Pesticide Detection

A general summary of the scaffolds used for fluorescence-based pesticide detection and the various advantages/disadvantages of each scaffold is shown in Table 1.

**TABLE 1:** Comparison of the various supramolecular architectures used for fluore**s**cence-based pesticide detection and the notable advantages/disadvantages of each method.

Supramolecular scaffold	Range of LODs	Significant advantages	Notable drawbacks
Polymers	1.3E-6 µM^137^–100 µM^126^	Conjugated fluorescent polymers have extremely high sensitivity; MIPs have high selectivity	Synthetic procedures can be cumbersome; organometallic polymers can be toxic and/or have toxic degradation products
Macrocycles	1E-6 µM^211^–1,040 µM^297^	Ability to rationally design components of the macrocycle to achieve target application	Synthetic procedures are nearly always cumbersome; can have poorly defined cavity for analyte binding
Nanomaterials	1.14E-7 µM^367^–12.2 µM^350^	High sensitivity, selectivity, and stability in a broad range of environments	Toxicity of the metal components can be of concern; as can the degradation and disposal of such materials
Metal-organic frameworks	5.2E-6 µM^413^–2.6 µM^420^	Easy and highly modular synthesis; large cavities able to bind multiple pesticide analytes	Limited stability in aqueous media; limited options for post-synthetic modifications

### Fluorescent Polymer-Based Pesticide Detection

Polymer-based sensors are well-established for a variety of analytes, including pesticides, using a range of structures. This section of the review is divided by type of polymer, including conjugated fluorescent polymers, molecularly imprinted polymers, and organometallic polymers. Each of these categories will be discussed in turn.

i. Conjugated fluorescent polymers. Conjugated polymers as fluorescence sensors for small molecule analytes were popularized by Swager and co-workers in the late 1990s, with their report that a conjugated fluorescent polymer acted as an effective molecular wire to facilitate ultrasensitive TNT detection (Yang and Swager, 1998a; Yang and Swager, 1998b). Extensive work since that initial report by Swager (Thomas et al., 2007; Rochat and Swager, 2013) and others (Liu and Bazan, 2007; Hergert et al., 2018) has demonstrated the broad applicability of this platform, based on the ability of the conjugated polymer to facilitate electronic communication throughout the length of its backbone. The performance of conjugated polymer sensors in solution has been well-established, and even greater performance has been observed in solid-state sensors (Joly et al., 2006) and in aggregated polymer nanoparticles (Ahmed et al., 2013); through the use of lasers (Rose et al., 2005) for targeted two-photon excitation (Goswami et al., 2020); through the incorporation of fluorescent polymers into sensor arrays for accurate analyte identification (Tropp et al., 2020); and through the inclusion of conjugated polymers as components of carbon nanotube-based sensors (Vu et al., 2020).

**FIGURE 3:**
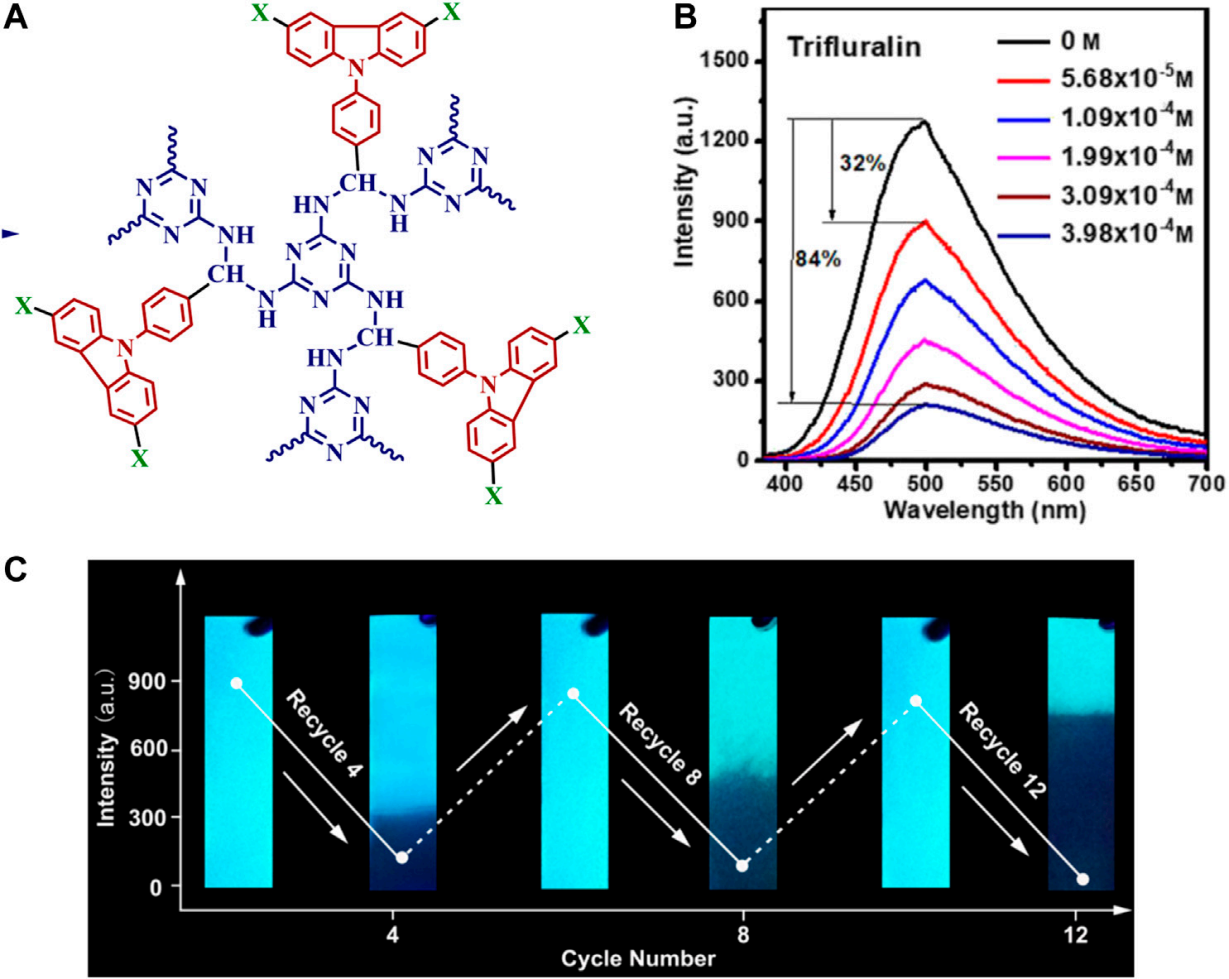
**(A)** Structure of the highly porous fluorescent carbazole-containing polymer used for pesticide detection **(B)** Illustration of pesticide-induced fluorescence changes in the carbazole-containing polymer, with the addition of trifluralin leading to an 84% decrease in the observed fluorescence emission; and **(C)** Photographs of filter papers functionalized with the carbazole-containing polymer and how they can be reused for multiple solid-state detection cycles. Reprinted from reference Zhang et al. (2020a).

The use of fluorescent polymers for pesticide detection has been reported by a number of research groups (Hao et al., 2019), in many cases relying on the ability of pesticides to act as fluorescence quenchers. In one example, Zhang et al. reported that a carbazole-containing polymer with significant inherent porosity was able to bind six pesticides in its porous structure, with the magnitude of fluorescence quenching different for each pesticide investigated (Figure 3A) (Zhang et al., 2020a). Of note, this detection operated both in solution (Figure 3B) and on papers onto which the polymer was adsorbed, with multiple detection-washing-reuse cycles reported (Figure 3C). The authors hypothesize that the porosity of the polymer is critical in enabling the pesticides to bind in close proximity to the polymer, in agreement with the work of Swager and co-workers that demonstrated significant benefit to free space around the polymer backbone (Swager, 2008). In another example of pesticide-induced fluorescence quenching, poly(2,6-dimethoxynaphthalene), which was electrochemically synthesized, responded to the presence of the pesticide imidacloprid with significant fluorescence quenching (Lei et al., 2013). Finally, a third example of pesticide-induced fluorescence quenching used a highly porous polyaminal structure, which was quenched by the addition of pesticide analytes (Zhang et al., 2018). All three of these examples likely proceed *via* FRET in order to obtain the observed pesticide-induced fluorescence quenching.

A different mechanism of pesticide detection using fluorescent polymers was reported by Levine and co-workers, who demonstrated that the introduction of pesticides to conjugated polymer nanoparticles and thin films (derived from 2,1,3-benzooxadizole-*alt*-fluorene) led to an increase in the fluorescence emission (Talbert et al., 2016). This result was attributed to the formation of reversible charge-transfer complexes between the polymer and the organochlorine pesticides that required electronic communication between the polymer chains, and was highly specific for organochlorine containing analytes. Finally, some degree of regeneration (up to 11 cycles) was demonstrated in this system, using iodine vapor to remove the pesticide and restore the original polymer fluorescence, although a moderate decrease in the fluorescence signal was observed with each cycle.

Pesticide detection using fluorescent conjugated polymers has also been reported to occur *via* analyte-induced deaggregation. In one example, Chen et al. reported that an amphiphilic monomer underwent free-radical polymerization in the presence of a cationic surfactant to generate a highly amphiphilic and luminescent polymer (Chen et al., 2019a), which aggregated in aqueous buffer to form luminescent nanoparticles. When combined with gold nanoparticles, the resulting construct responded to the presence of paraoxon with a decrease in the observed fluorescence emission consistent with analyte-induced deaggregation. Of note, this system worked in real-world samples to detect paraoxon in contaminated lake water and cabbage extract, and to recover the paraoxon with high efficiency (>93%).

Indirect pesticide detection was also reported through monitoring the enzymatic activity of acetylcholinesterase (AChE), based on the ability of several pesticides to act as effective AChE inhibitors. One example using AChE inhibition was reported by Han et al., based on FRET which occurred from fluorescent conjugated polymer nanoparticles to manganese oxide (MnO_2_) nanosheets that was disrupted in the presence of AChE (Han et al., 2019). Carbaryl, a pesticide which inhibits AChE activity, limited AChE-induced nanosheet destruction and restored the fluorescence signal. Other examples of using AChE activity as a biomarker for the presence of pesticides have also been reported (Nikoleli et al., 2011; Jin et al., 2019; Ma et al., 2020).

A final example of conjugated polymer-based pesticide detection was reported by Chen and coworkers (Chen et al., 2020a). In this example, negatively charged fluorescent nanoparticles were combined with gold nanoparticles that were coated with a cationic conjugated fluorescent polymer. The resulting electrostatic attraction between the two components led to a self-assembled supramolecular architecture, whose fluorescence was enhanced in the presence of the pesticide analyte, malathion. This resulted in highly sensitive malathion detection (limit of detection at 1.42 nM) as well as a high degree of selectivity for the malathion compared to other analytes investigated.

In general, the key advantage of using conjugated fluorescent polymers for pesticide detection is the high sensitivity of such polymers (Xu et al., 2004). This sensitivity has been well-documented in general (Rasheed et al., 2020), and has also been demonstrated in the context of highly sensitive pesticide detection (Zhou et al., 2017). Additionally, effective conjugated polymer-based detection can occur under a broad variety of conditions, including in aqueous solution, on solid-state devices, and in complex, real-world environments (Nsibande and Forbes, 2019). Key disadvantages focus on the fact that the synthesis and purification of conjugated polymers is often complex (Swager, 2017), and that the lack of straightforward access to such polymers can preclude their widespread usage.

ii. Molecularly imprinted polymers. The concept of molecularly imprinted polymers (MIPs) has origins that span several decades (Samarth et al., 2015), and was formalized in the 1980s, when Wulff (Wulff et al., 1986) and Mosbach (Andersson and Mosbach, 1990) reported that polymerization in the presence of small molecule substrates led to improved performance in chromatography (Andersson et al., 1990), enantiomeric resolutions (O'Shannessy et al., 1989), and catalysis (Robinson and Mosbach, 1989). In general, molecular imprinting involves polymerization that occurs in the presence of a template, which is bound covalently or non-covalently to the polymer scaffold (BelBruno, 2019). Removal of the template after polymerization results in a structure with sites that are tailored to bind an analyte which is similar to the template (Xu et al., 2019b). MIPs have found success in a variety of applications, including in chromatography (Bereli et al., 2020), sensing (Wang et al., 2020d), and catalysis (Muratsugu et al., 2020).

Moreover, MIPs have enjoyed success as components of fluorescent pesticide sensors. In a relatively early example, carbaryl was used as a template for the photochemically induced polymerization of acrylamide to generate MIPs with carbaryl binding sites (Sanchez-Barragan et al., 2007). The removal of carbaryl after polymerization occurred through Soxhlet extraction, which removed approximately 80% of the template. The resulting structure was able to bind exogenous carbaryl with high efficiency, enabling effective fluorescence-based detection of the carbaryl analyte *via* binding-enhanced fluorescence emission of the carbaryl. Moreover, high levels of selectivity for carbaryl compared to other analytes were also observed, and were attributed to successful carbaryl-induced imprinting.

Another example occurred through the use of fluorescent MIP microspheres, which were synthesized *via* precipitation polymerization of a mixture of monomers, including allyl fluorescein, in the presence of the cyhalothrin analyte as a template (Gao et al., 2014). Moreover, because the template was not covalently attached to the polymer, removal of the template was straightforward. The resulting structure displayed both high sensitivity (attributed to FRET from the fluorescent microspheres to the nonfluorescent cyhalothrin) and selectivity (attributed to the imprinting effect) for cyhalothrin, and was used to detect the analyte in real-world honey samples.

Even greater sensor performance occurred when MIPs were attached to a solid support. In one example, an MIP was used as an integral component of lateral flow test strips for triazophos detection (He et al., 2020a). Detection was accomplished by competing the target analyte with a biological fluorescent probe composed of a triazophos hapten conjugated to both murine IgG and fluorescein. Notably, the optimized system provided a limit of detection of triazophos in tap water of 20 μg/L, while demonstrating high selectivity, reproducibility, and robustness in real-world environments. In another example, rotational paper-based microfluidic devices were fabricated using a molecularly imprinting polymer technique to facilitate the effective detection of nitrophenol-containing analytes (Qi et al., 2018), known pesticide precursors, side products, and decomposition products (Ren et al., 2021). Finally, combining MIPs with CdTe quantum dots on paper-based microfluidic chips led to a detection system for 2,4-dichlorophenoxyacetic acid with limits of detection as low as 90 nM that operated in both soybean sprouts and lake water samples (Zhang et al., 2020d).

Overall, while significant success using MIPs for pesticide sensors has been demonstrated, some disadvantages remain. These disadvantages include the fact that the synthetic effort required to access MIPs can often provide a logistical barrier, although strategies to accomplish effective syntheses have also been reported (Yang et al., 2018b). Other practical concerns relate to the ability to remove the template completely following polymerization, and the suitability of implementing such conditions in large scale sensor development. Notable advantages to the use of MIPs include their extremely high selectivity for the target analyte, which is accomplished by use of the template that creates perfectly tailored binding sites (Wang et al., 2020k), as well as their durability under a variety of conditions that enable widespread usage (Saylan et al., 2017).

iii. Organometallic polymers. Metal-containing organic polymers, or organometallic polymers (Nguyen et al., 2018), have a long history for use in sensing (Kumar et al., 2020a), optoelectronics (Onge et al., 2020), and catalysis (Joshi et al., 2020). They have been used as sensors for a variety of analytes (Xu et al., 2017), including pesticides, using a range of transduction mechanisms, including electrochemical (Wang et al., 2020g), colorimetric (Scarano et al., 2019), and Raman spectroscopy-based detection (Yan et al., 2019).

Such scaffolds have also been used for fluorescence-based pesticide detection with high levels of success. In one example, a cerium-based fluorescent polymer was combined with nanoparticles made of a samarium and cerium oxide composite to generate a supramolecular architecture, whose fluorescence emission was enhanced by aggregation induced by small molecule chelators (either adenosine triphosphate or Tris buffer) (Wei et al, 2020a). The resulting construct detected the pesticide methyl paraoxon (Petroianu et al., 2012) *via* pesticide-induced deaggregation of the supramolecular structure and concomitant fluorescence quenching. Of note, a strong pH dependence was also observed, consistent with supramolecular electrostatic interactions being the predominant underlying force that brings the sensor and pesticide into close proximity to enable effective energy transfer and sensitive pesticide detection. Reasonable detection limits for the analyte were determined (1.0 µmol/L), although other detection systems for methyl paraoxon have been reported with higher sensitivity (Wang et al., 2020e; Luo et al., 2020).

In an intriguing example, Jin et al. recently reported that organometallic hydrogels, composed of *o*-phenylenediamine, silicon quantum dots, and silver cations, could function as on-site pesticide sensors using smartphones for signal read-out (Jin et al., 2019). Introduction of organophosphate pesticides to these hydrogels results in inhibition of the enzyme AChE, which blocks the formation of thiocholine and the formation of an organometallic self-assembled polymer. Moreover, these changes cause oxidation of *o*-phenylenediamine to diaminophenazine, which corresponds to a strong change in the fluorescence signal. This signal change is due to the inner filter effect, in which the diaminophenazine absorbs excited state energy and limits the energy that reaches the silicon quantum dots.

**FIGURE 4:**
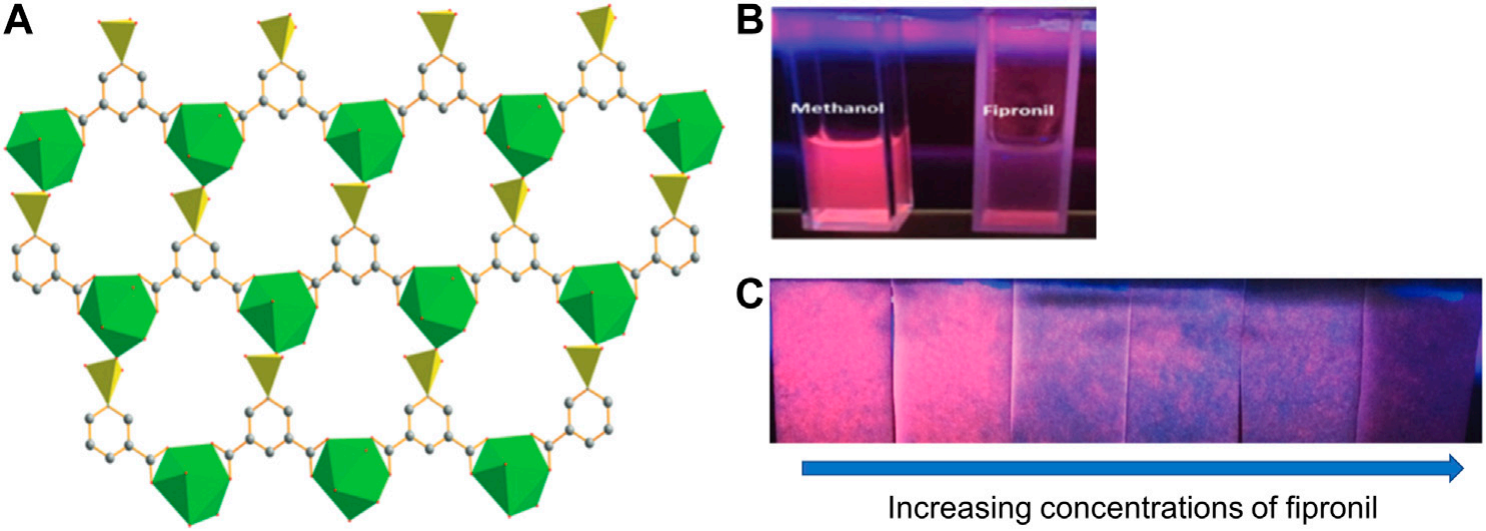
**(A)** Depiction of the X-ray crystal structure of the two-dimensional europium coordination polymer. The green polyhedral represent the europium-oxygen coordination polymer and the yellow triangles represent the SO_3_C tetrahedron centered at the S atoms **(B)** Photograph of methanol solutions containing the europium coordination polymer in the absence (left side) and presence (right side) of the pesticide fipronil; and **(C)** Photograph of filter papers treated with the europium coordination polymer and illuminated with a 254 nm excitation source in the presence of increasing concentrations of fipronil. Reprinted from reference Yang et al. (2018c).

One final example is the fluorescence-based detection of fipronil (Li et al., 2020c), a small molecule pesticide that was reported as a contaminant in European eggs (Yang et al., 2018c). Detection was accomplished with a europium-containing coordination polymer, which displayed a strong fluorescence emission that was quenched in the presence of fipronil (Figure 4). Significant overlap in the absorption spectrum of fipronil and the excitation bands of the fluorescent detector indicate the likelihood of fipronil acting as an inner filter to attenuate the energy that reach the luminescent europium polymer. This system operated both in solution (Figure 4B) and on papers on which the fluorescent organometallic polymer had been adsorbed (Figure 4C). Moreover, this system displayed remarkably high selectivity, with other structurally similar pesticides leading to virtually no fluorescence changes. Finally, the limit of detection for the analyte was 0.8 µM, which is close to the maximum contamination levels reported by European (European Food Safety Authority Reasoned, 2014) and United States (Nougadere et al., 2011) regulatory authorities.

Like the conjugated fluorescent polymers and molecularly imprinted polymers discussed in the previous two sections, organometallic polymers are known to provide high levels of sensitivity, which constitutes a significant advantage of their usage (Fegley et al., 2012). Moreover, many of these structures are fabricated using metal-ligand directed self-assembly, which is often simpler than fully organic syntheses (Yan et al., 2011). A downside to such polymers is that the metal components can have non-trivial toxicity throughout their life cycle: in the starting materials, in the organometallic polymer sensor, and in the degradation products of the polymer after usage (Hudson and Katz, 2018); this toxicity constitutes a significant potential disadvantage. Moreover, while many of the metal-ligand coordination bonds are stable both in solution and in the solid-state, they are overall weaker compared to covalent bonds, and the potential for disassembly under extreme circumstances remains (Bao et al., 2014).

iv. Polymers combined with other supramolecular structures. The combination of polymers with other supramolecular structures has, in many cases, led to enhanced performance and detection capabilities. In one example, MIPs were combined with silica particles that contained embedded CdSe nanospheres to yield highly fluorescent nanostructures, (Sun et al., 2011). Binding of parathion methyl, the target pesticide, to the MIP resulted in a noticeable decrease in the fluorescence emission as a result of proximity-induced fluorescence quenching. Of note, in addition to demonstrating high sensitivity and selectivity, the sensor was able to detect parathion methyl in real-world vegetable samples in which the target analyte had been doped.

Another intriguing example was reported by Liu and co-workers, who used a Pickering emulsion polymerization to develop supramolecular architectures that contained a molecularly imprinted polymer, highly luminescent europium complex, and silica nanospheres (Liu et al., 2013). The resulting constructs, following removal of the small molecule template, were used to detect λ-cyhalothrin with high selectivity *via* analyte-induced fluorescence quenching. Moreover, the ratiometric nature of this system means that quantification of unknown analyte concentrations is theoretically possible.

A third example of using MIPs with other supramolecular constructs was reported by Wei et al. in 2015 (Wei et al., 2015). Luminescent CdTe quantum dots were combined with MIPs that had been synthesized in the presence of a bifenthrin template to yield a composite pesticide sensor, in which bifenthrin binding led to markedly decreased fluorescence emission, with low limits of detection and high levels of selectivity reported as well. The novelty of this system lies in the synthetic approach, which uses a biphasic solvent system to induce polymerization on the CdTe quantum dot surfaces. Removal of the non-covalently attached template in this case occurred via a simple solvent extraction. Finally, a broad variety of other MIP-nanomaterial constructs for luminescent pesticide sensing have also been reported (Chen et al., 2018b), and the interested reader is directed towards these references for further information (Li et al., 2017; Wang et al., 2017; Huang et al., 2018).

Overall, advantages of using these combination architectures include the fact that such combinations can provide properties that are not accessible to either component alone. For example, the combination of MIPs with a CdTe quantum dot core led to a material that could detect nitrophenol in a broad variety of complex environments (Yu et al., 2017), and related systems have also been reported (Wang et al., 2016b). Disadvantages center around the complex preparations required for such architectures, which include generally separate preparations of each component prior to assembly of the final structure. Nonetheless, these combination structures retain the potential for significant performance enhancements in practical pesticide detection.

### Macrocycle-Based Pesticide Detection

The fluorescence-based detection of pesticides using macrocycles can occur in two ways: 1) a non-fluorescent macrocycle acts as a scaffold to bring a pesticide in close proximity to a fluorescent signaling element or to amplify the pesticide’s innate fluorescence; or 2) a fluorescent macrocycle responds to the presence of the pesticide with a change in its emission. Each of these options will be discussed in turn.

Non-fluorescent macrocycles including cyclodextrins, cucurbiturils, calixarenes, pillararenes, and synthetic macrocycles, will be discussed herein.

#### Cyclodextrins

**FIGURE 5:**
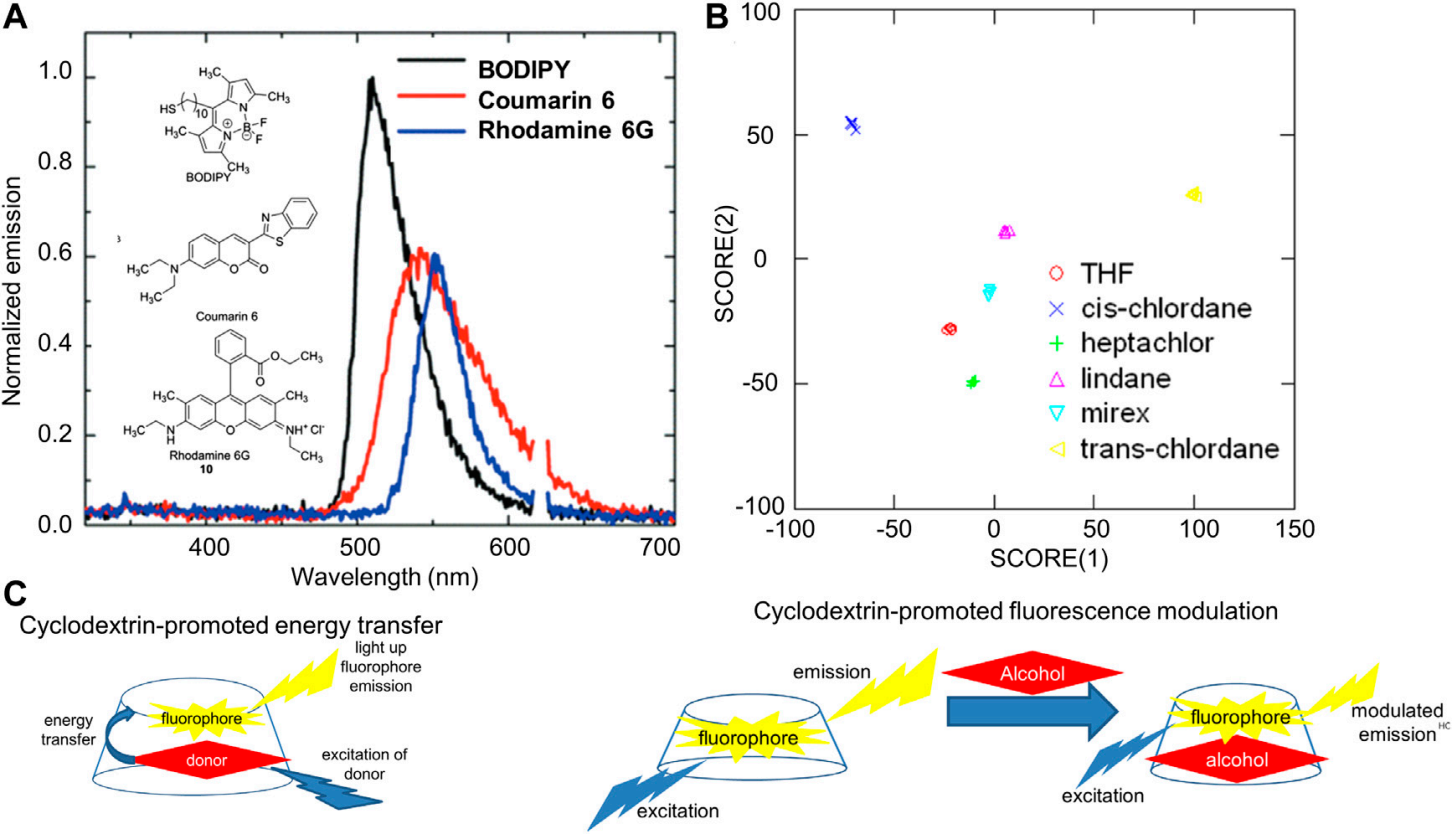
**(A)** Illustration of fluorescence emission spectra resulting from cyclodextrin-promoted energy transfer from pesticide deltamethrin to BODIPY, rhodamine, and coumarin fluorophores (reprinted from reference Serio et al. (2015a)) **(B)** Illustration of how differential pesticide-induced fluorescence responses in Arcadia Lake (a freshwater lake in Rhode Island) can be used to achieve differentiation in linear discriminant analysis (reprinted from reference DiScenza et al. (2017); and **(C)** General depiction of cyclodextrin-promoted fluorescence energy transfer and cyclodextrin-promoted fluorescence modulation (reprinted from reference DiScenza and Levine (2016b).

The use of cyclodextrins as scaffolds that can promote interactions between pesticide analytes and a fluorescent signaling element was reported by Levine and co-workers in 2015, in which γ-cyclodextrin promoted proximity-induced interactions between aromatic pesticides and high quantum yield fluorophores, leading to fluorescence energy transfer from the pesticide to the fluorophore (Figure 5A) (Serio et al., 2015a). Limits of detection and quantification calculated were as low as 2.1 µM in the complex environment of apple juice solution (selected as a matrix due to reports that such solutions can have high pesticide concentrations) (Zhang et al., 2015b).

This work was expanded to include non-aromatic pesticide detection *via* cyclodextrin-promoted, analyte-specific changes in the emission of a high quantum yield fluorophore held in close proximity to the pesticide, leading to a dramatic expansion in the scope of pesticides detectable *via* this method (DiScenza and Levine, 2016a). Subsequent work demonstrated that the same principle of analyte-induced fluorescence modulation could be used to detect non-aromatic pesticides in real-world water samples taken from a locations within Rhode Island, with the high analyte specificity easily translatable to 100% selectivity *via* linear discriminant analysis (Figure 5B) (DiScenza et al., 2017). More generally, this process, termed “fluorescence modulation” (Figure 5C) (DiScenza and Levine, 2016b), expanded the scope of non-pesticide analytes detectable *via* cyclodextrin-promoted noncovalent interactions (Serio et al., 2013; Serio et al., 2015b) to include non-aromatic analytes as well (DiScenza et al., 2018; DiScenza et al., 2019).

Other examples of fluorescence-based detection of pesticides using cyclodextrins have also been reported, including those that use binding in cyclodextrin to enhance the fluorescence emission of weakly fluorescent carbamate pesticides (Pacioni and Veglia, 2007; Ogoshi and Harada, 2008). The fluorescence of these pesticides, bendiocarb and promecarb, increased in the presence of β-cyclodextrin or γ-cyclodextrin between 1.74 and 3.8-fold, enabling fluorescence detection. Other related work using cyclodextrin to enhance the innate pesticide fluorescence has also been reported (Marquez et al., 1990; Coly and Aaron, 1998; Wagner et al., 2002; Pacioni and Veglia, 2003).

In another example, researchers compared the fluorescence enhancement of pesticides in the presence of two macrocyclic hosts: *p*-sulfonatocalix [6]arene and cyclodextrins (Pacioni et al., 2008). Strong electrostatic interactions were observed between the cationic pesticides and anionic *p*-sulfonatocalix [6]arene, with weaker hydrophobically driven binding observed for the pesticides in neutral α-cyclodextrin and 2-hydroxypropyl-β-cyclodextrin (2-HPCD). Despite these differential binding affinities, however, the greatest binding-induced fluorescence amplification was observed for 2-HPCD (Table 2), measured as the ratio of the quantum yield in the presence of the complex to the quantum yield of the free pesticide, and defined according to Eq. 1, below:

$$\text{Relative}\;\text{fluorescence}\;\text{quantum}\;\text{yield}\;\text{ratio}={\text{QY}}_{\text{complex}}/{\text{QY}}_{\text{free}}.$$ (1)

where *QY*_complex_ refers to the relative quantum yield of the pesticide in the presence of the supramolecular complex, and *QY*_free_ refers to the relative quantum yield of the pesticide in the absence of any supramolecular host.

**TABLE 2:** Relative fluorescence quantum yield ratios and binding constants reported for pesticide benomyl in the presence of supramolecular hosts^a^.

Host identity	K_a_ (M^−1^)	Relative quantum yield ratio^b^
α-Cyclodextrin	(17 ± 3) x 10^1^	1.40 ± 0.03
2-Hydroxypropyl-β-cyclodextrin	(3 ± 1) x 10^1^	2.8 ± 0.4
*p*-Sulfonatocalix [6]arene	(2.6 ± 0.4) x 10^5^	2.00 ± 0.05

^a^ All results were obtained in aqueous solution at a pH of 1.0 and at 25°C.

^b^ Relative quantum yield ratios were calculated as the ratio of the relative quantum yield of the pesticide in the complex divided by the relative quantum yield of the free pesticide, according to Eq. 1.

Results in Table 2 highlight the superior performance of 2-HPCD, with the highest observed fluorescence enhancements and lowest limits of detection. Moreover, 2-HPCD was used to recover the pesticides from complex samples with near quantitative (91–106%) pesticide recovery. The significant benefits of 2-HPCD can be explained based on the high flexibility (Mokhtar et al., 2018), good solubility (Wu et al., 2017a), and strong hydrophobically driven complexation that is known for 2-HPCD in a variety of complexation scenarios (Geng et al., 2019).

Other options for fluorescent detection using cyclodextrins include attaching a fluorescent moiety to the cyclodextrin core, with binding of pesticides to such modified structures resulting in changes in the emission of the fluorescent substituent. For example, Chaudhuri et al. reported that coumarin-functionalized β-cyclodextrin bound a variety of small molecule analytes, including DDT and related analogues, with highly analyte-specific changes to the fluorescence emission observed (Chaudhuri et al., 2017). This system resulted in 100% differentiation between the fluorescent signals corresponding to structurally similar analytes with micromolar limits of detection. The notably high selectivity was attributed to the covalent attachment of coumarin, which facilitated close-range interactions between the analyte and fluorophore.

**FIGURE 6:**
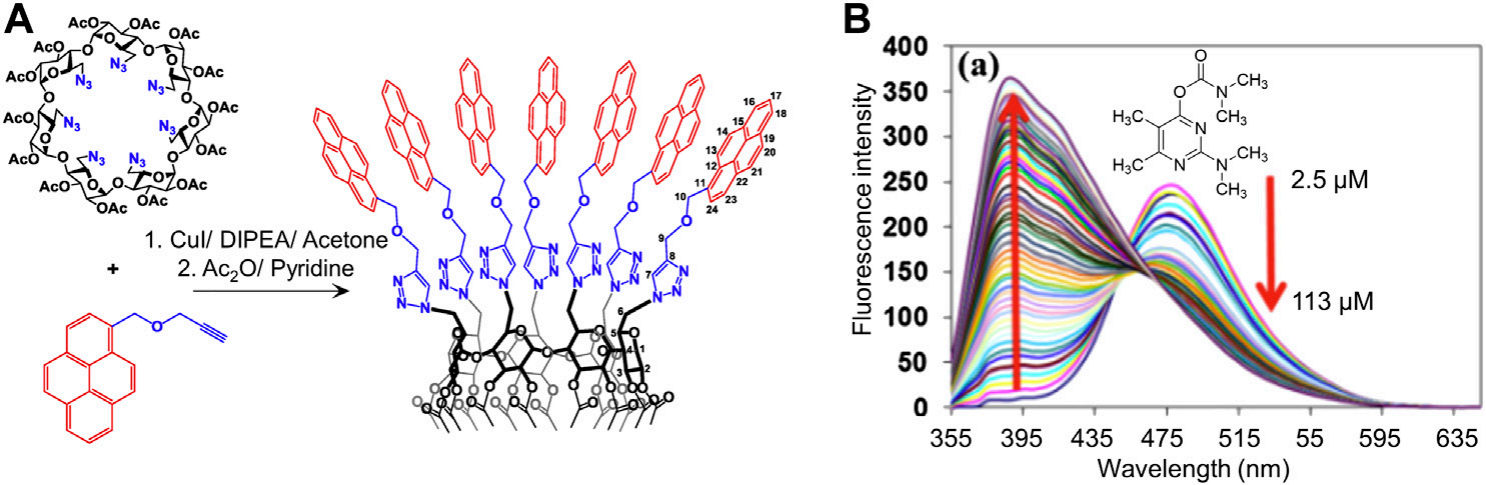
**(A)** Synthesis of pyrene-appended β-cyclodextrin *via* a Huisgen cycloaddition reaction; and **(B)** Changes in the fluorescence intensity of pyrene-appended β-cyclodextrin with the addition of increasing concentrations of pirimicarb, showing an increase in pyrene’s monomer emission and decrease in its excimer emission. Reprinted from reference Champagne et al. (2019).

In another example, researchers reported that pyrene-appended β-cyclodextrin forms fluorescent aggregates in aqueous solution (Figure 6) (Champagne et al., 2019). Such aggregation is disrupted by the pesticide pirimicarb, leading to a decrease in the aggregate emission and an increase in the monomer emission that facilitates ratiometric pesticide detection.

In a different example, a fluorescent aromatic core bound three cyclodextrin moieties, with Huisgen dipolar cycloaddition reactions used to covalently link all components (Mallard-Favier et al., 2009). The resulting cyclodextrin trimer was used for the fluorescence-based detection of pesticides using pesticide-induced changes in the structure’s fluorescence emission. One drawback to this work, however, is that the complexation and detection occurred only in acetonitrile; applications of such a system in purely aqueous solution (and in complex aqueous environments) has not yet been demonstrated.

In addition to adding fluorescent substituents to the cyclodextrin scaffold, researchers have also attached luminescent metal substituents to obtain fluorescent sensors. In one example, an amino-substituted β-cyclodextrin complexed to europium (III) responded to the presence of fenitrothion with enhanced long-wave luminescence. Notably, the high selectivity observed for the fenitrothion analyte compared to other pesticides was attributed to the selectivity of β-cyclodextrin in binding small molecule guests (Kanagaraj et al., 2012).

Other cyclodextrin-based fluorescent pesticide sensors include those in which cyclodextrin is combined with nanorods (Luo et al., 2015a), quantum dots (Li and Han, 2008), and molecularly imprinted polymers (Farooq et al., 2020). Overall, significant practical advantages of using these supramolecular constructs have been noted.

In general, cyclodextrin-based pesticide detection builds on the known ability of cyclodextrin to bind pesticides (Marican and Duran-Lara, 2018), which is often used to remove pesticides from contaminated environments (Flaherty et al., 2013). Cyclodextrin-mediated environmental remediation of pesticides occurs effectively in contaminated soils (Wong and Bidleman, 2010), sediments (Yang et al., 2010), and water (Liu et al., 2011), with both unmodified and synthetically modified cyclodextrin hosts (Sawicki and Mercier, 2006). Finally, interactions between cyclodextrins and pesticides have also been used for non-fluorescence-based pesticide detection, including *via* electrochemical changes (Hromadová, et al., 2002), Raman spectroscopy (Strickland and Batt, 2009), and electroluminescence signaling (Wang et al., 2016).

Cyclodextrin-based sensors have a number of notable advantages compared to other methods, including the ability to use a non-toxic, water soluble, and commercially available molecule to accomplish pesticide detection. The high flexibility of cyclodextrin, in turn, can either benefit the cyclodextrin-based sensors, through enabling them to bind a range of pesticides, or harm the cyclodextrin-based sensor performance, through reducing selectivity for a particular pesticide target (Aleem et al., 2020). Moreover, the high flexibility of the cyclodextrin scaffold can complicate efforts to fully characterize pesticide-cyclodextrin binding affinities, geometries, and stoichiometries (Dodziuk, 2002). The broad-based utility of cyclodextrin-based sensors, in purified aqueous solutions, in real-world contaminated water samples, and when adsorbed covalently or non-covalently to a solid surface, further enhances cyclodextrin’s general applicability and overall sensor potential (Tsuchido et al., 2017).

#### Cucurbiturils

Like cyclodextrin, cucurbiturils are supramolecular hosts capable of binding a variety of small molecule guests (Masson et al., 2012). They differ from cyclodextrins in the rigidity of their structures, in their high structural symmetry, and in the existence of electronegative carbonyl groups that line the cavity portal (Isaacs, 2009). As a result of such differences, cucurbituril binds small molecules with higher affinity than cyclodextrin (due to the increased rigidity in their structures) (Lagona et al., 2005), and are particularly well suited to bind guests that are both cationic (to associate with the carbonyl groups) (Huang et al., 2008) and hydrophobic (to associate with the hydrophobic cavity) (Nau et al., 2011).

**FIGURE 7:**
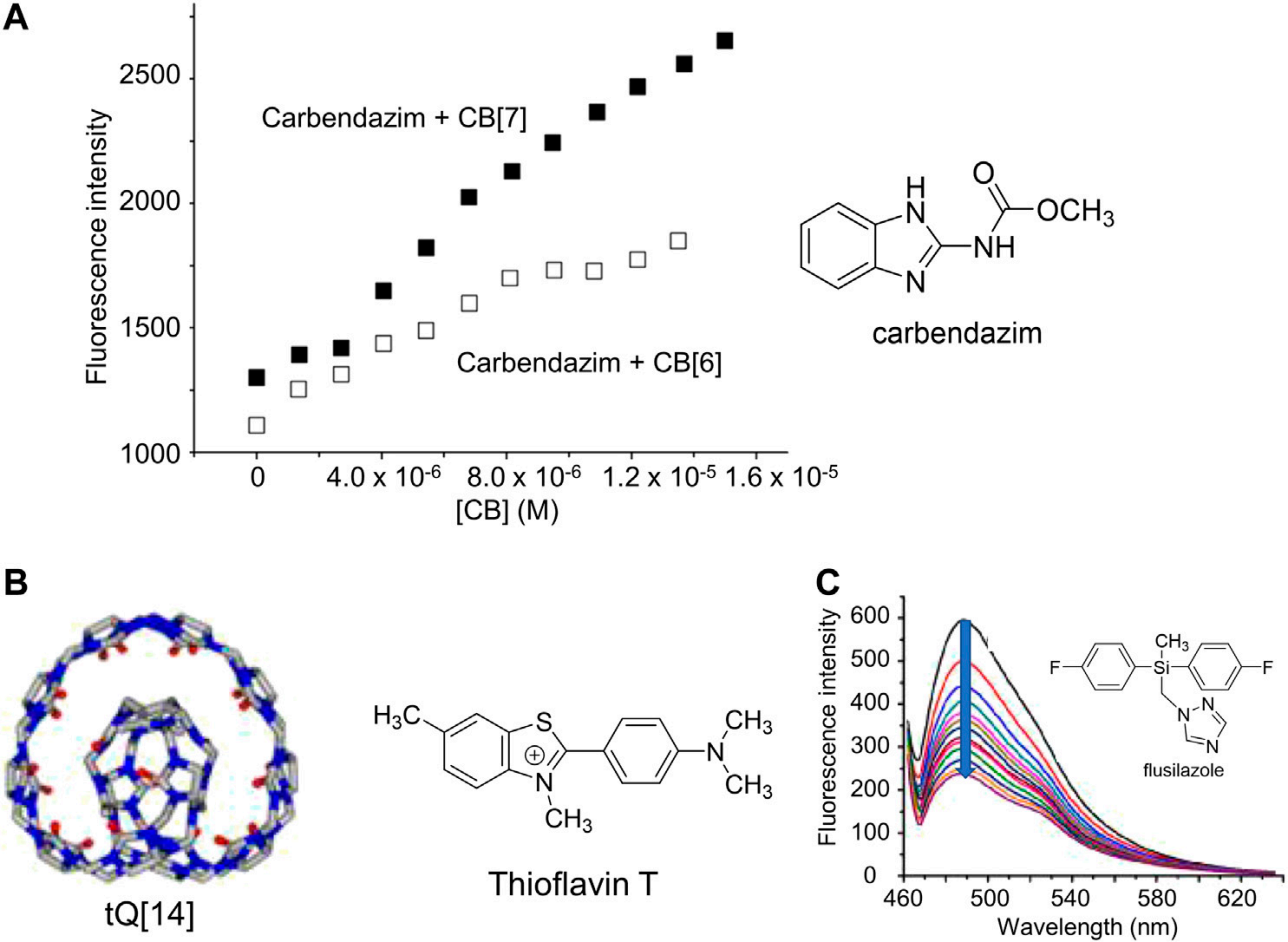
**(A)** Changes in the fluorescence intensity of carbendazim as a function of increasing concentrations of cucurbit [6]uril (CB [6]) and cucurbit [7]uril (CB [7]). 0.2 M acetate buffer; pH = 4. *λ*_ex_ = 298 nm; *λ*_em_ = 305 nm. Reprinted from reference del Pozo et al. (2010) ((**B)** Structure of the twisted cucurbit [14]uril (tQ [14]) and thioflavin T used as the fluorescent probe; and **(C)** Illustration of changes in the fluorescence spectra of the tQ [14]-thioflavin T complex with the introduction of fungicide flusilazole (*λ*_ex_ = 488 nm) Reprinted from reference Fan et al. (2019).

Like cyclodextrins, cucurbiturils that bind pesticides often increase the native pesticide emission, enabling direct fluorescence detection. Moreover, because cucurbiturils have higher binding affinities for pesticides (Assaf and Nau, 2015), the concomitant fluorescence increases are often higher as well. For example, cucurbit[7]uril forms a strong supramolecular complex with the pesticide carbendazim, resulting in increased pesticide fluorescence emission that enables its monitoring in real-world samples (Figure 7A) (del Pozo et al., 2010). Detailed computational work on this system determined that charge transfer between the carbendazim and cucurbit[7]uril was the dominant mechanism in enabling the fluorescence enhancement to occur, which led to extremely low limits of detection and quantification (0.10 and 0.52 mg/kg, respectively) (Fatiha et al., 2015).

Further research established the general applicability of cucurbituril-enhanced fluorescence emission for pesticide detection (Elbashir and Aboul-Enein, 2015). In another example, thiabendazole bound to a variety of cucurbiturils in aqueous solution, which resulted in enhanced thiabendazole emission and nanomolar-level detection of this analyte (Huang et al., 2012). Interestingly, ^1^H NMR studies established that the binding geometry differed depending on the identity of the cucurbituril, with cucurbit[7]uril selectively binding the benzimidazole ring and cucurbit[6]uril binding the thiazole ring, as a result of differences in their cavity dimensions (Liu et al., 2015). Other cases in which cucurbituril binding results in increased pesticide fluorescence emission and concomitant fluorescence-based detection have also been reported (Saleh and Al-Rawashdeh, 2006; Jing et al., 2012).

An interesting example was seen in the detection of the pesticide paraquat (Chen et al., 2010; Vaccari et al., 2017; Kim and Kim, 2020), which is also a well-known fluorescence quencher (Schibilla et al., 2016; Willis-Fox et al., 2016). Detection of paraquat using cucurbiturils was accomplished through first forming a complex between cucurbituril and acridine orange, resulting in enhanced fluorescence of the acridine orange dye (Xing et al., 2013). Subsequent addition of paraquat caused a displacement of the dye from the cucurbituril cavity due to the high binding affinity of paraquat for cucurbituril, and formation of a cucurbituril: paraquat supramolecular complex, resulting in overall fluorescence quenching. Of note, the strong binding affinity of paraquat in cucurbituril is attributed to the affinity between the positively charged nitrogen atoms in paraquat and the electronegative carbonyl oxygens that line the cucurbituril rim (Kaifer, 2018).

In contrast, introduction of paraquat to a different cucurbituril system led to a fluorescence increase (Sun et al., 2014). In this case, cucurbit[8]uril bound two methylene blue molecules simultaneously, resulting in fluorescence quenching due to dimerization (Dean et al., 2016). Introduction of paraquat led to displacement of the small molecule guests from the cavity, like in the aforementioned case. In this case, however, displacement of methylene blue from cucurbituril destroyed the supramolecular dimer, resulting in restoration of the monomeric fluorescence emission.

Another example of using cucurbiturils for pesticide detection was reported by Cao and co-workers, in which fluorescent CdTe quantum dots were coated with cucurbiturils. The resulting hybrid construct bound *p*-nitroaniline, a known component of pesticides (Li et al., 2010), in close proximity to the quantum dot core, resulting in efficient fluorescence quenching. Advantages to this method include the low detection limits (6 × 10^−8^ M) and a decreased requirement to pretreat real-world samples prior to analysis. Disadvantages include the fact that other small molecule quenchers that bind in cucurbituril are likely to interfere with system selectivity.

In one final example, researchers reported the use of a twisted cucurbit[14]uril (tQ [14]) (Li et al., 2016), for fluorescence-based pesticide detection (Figures 7B,C) (Fan et al., 2019). This molecule bound thioflavin T in one part of its structure, resulting in markedly enhanced emission. Subsequent introduction of triazole-containing pesticides resulted in a strong fluorescence decrease. Detailed investigations revealed that two different supramolecular mechanisms were operative: one in which the triazole fungicide bound to a different section of tQ [14] than the thiazole dye, resulting in cooperative effects that facilitated the fluorescence decrease; and one in which the triazole fungicide and the triazole dye competed for the same binding site, also resulting in the observed fluorescence quenching.

Notable advantages to cucurbituril-based sensors include the extremely high binding affinity of such macrocycles, which have been shown to bind guests with attomolar-level dissociation constants (Cao et al., 2014). Such strong binding, in turn, enables high sensitivity in cucurbituril-based sensors, even within highly complex environments (Hennig and Nau, 2020). While the synthetic procedures to access cucurbiturils can be quite cumbersome, recent results from Isaacs (Lu et al., 2020b) and others (Liu et al., 2019; Bauer et al., 2019) have demonstrated that acyclic cucurbituril receptors can provide binding affinities that are nearly indistinguishable from their cyclized analogues; such acyclic analogues, in turn, are markedly easier to access.

#### Calixarenes

Calixarenes are defined as macrocycles with phenol-based monomers, linked through CH_2_ spacers to generate well-defined hydrophobic cavities (Rebek, 2000). They have more structural flexibility than cucurbiturils and cyclodextrins, and their aromatic character makes them particularly well-suited to bind aromatic guests (Brugnara et al., 2014; Yabushita et al., 2018).^,^ Calixarenes have been used extensively as chemical sensors (Sharma and Cragg, 2011), including as pesticide sensors. In one example, *p*-tert-butylcalix[4]arene was bound to the surface of ruthenium-containing silicon nanoparticles, with the resulting construct used to bind glyphosate in the calixarene cavities (Kier and Kirkland, 2013). Such binding resulted in fluorescence resonance energy transfer (FRET) and extremely sensitive glyphosate detection (limit of detection = 7.91 × 10^−7^ M) (Bosco et al., 2018). While the sensitivity of this system was extremely high, the requirement for ruthenium and silicon-based materials raises some questions about the long-term environmental sustainability of the reported system.

Other calixarene-containing constructs for pesticide detection include the use of calixarene coatings on CdTe quantum dots, which were included in silica spheres *via* a unique sol-gel process. The resulting supramolecular construct detected glyphosate *via* its binding in the calixarene cavities, resulting in fluorescence quenching (Li et al., 2012). Moreover, even in cases in which calixarene was not covalently attached to quantum dots, its presence in solution was sufficient to facilitate pesticide detection (Qu et al., 2009). In this example, the ability of calixarene found in solution to bind fenamithion and acetamiprid was sufficient to remove these pesticides from proximity to the quantum dots and restore the quantum dot’s fluorescence.

Finally, CdTe quantum dots embedded in a silica matrix that was coated with a calix [4]arene derivative bound the pesticide methomyl with marked increases in the observed fluorescence emission (Li and Qu, 2007). Importantly, the system in this case displayed highly selective binding for methomyl (Morse et al., 1979; Shalaby et al., 2010), with other pesticides, including parathion, fenamithion, optunal, and acetamiprid, causing no fluorescence changes. The authors hypothesize that the origin of the high selectivity may be due to the fact that methomyl triggers an ordered arrangement of the surface calixarenes, with limited analyte-induced distortion. Evidence in support of this hypothesis comes from the fact that system data fit well with a Langmuir isotherm model, indicating a highly ordered surface structure (Kostyukevych et al., 2016).

In another interesting example, Xu et al. reported that a chiral calixarene covalently attached to a gold surface bound naproxen enantioselectively, leading to fluorescence changes (Xu et al., 2019a). Moreover, the chirality of the calixarene was controlled photochemically, meaning that either naproxen enantiomer could be bound depending on experimental conditions. Of note, while naproxen is an over-the-counter pharmaceutical drug rather than a pesticide (Angiolillo and Weisman, 2017), it has been found concurrently with pesticides in many cases (Anim et al., 2020; Bommuraj et al., 2020), and can provide important information about the overall water contamination.

Additionally, much like cyclodextrins and cucurbiturils, calixarenes that bind weakly fluorescent pesticides often cause increases in the pesticide’s emission. In one example, calix [4]arene and 4-*tert*-butyl-calix[6]arene were investigated as hosts for benzotrifluoride, a component of pesticides (Manfrin et al., 2020). The binding of benzotrifluoride in the calixarenes led to noticeable fluorescence increases (Kunsagi-Mate et al., 2001), with the calixarenes binding more strongly than comparable cyclodextrin hosts (10^5^ M^−1^ in calixarenes compared to 10^1^–10^2^ M^−1^ for the cyclodextrins), leading to a 2-fold increase in the relative quantum yield of the pesticide (Pacioni et al., 2008).

Resorcinarenes, a class of macrocycles that are related to calixarenes (formed from resorcinol monomers rather than phenol monomers), have been used occasionally for pesticide detection. In one example, a tetra-sulfonated resorcinarene was attached to a silver nanoprobe via non-covalent adsorption (Menon et al., 2013), leading to a system that responded to the pesticide dimethoate with notable fluorescence and colorimetric changes. The proposed mechanism for dimethoate inclusion and for the observed selectivity focused on the fact that dimethoate has two polar termini, each of which can bind to a resorcinarene, resulting in analyte-induced aggregation of the silver nanoparticles and concomitant spectroscopic changes.

Of note, in addition to using calixarenes for fluorescence-based pesticide detection, researchers have used calixarenes in other detection modes (Sanabria Espanol and Maldonado, 2019). This includes electrochemical detection to monitor the binding of heptachlor in 4-sulfocalix[6] arene (Bocchinfuso et al., 2008); Raman signaling to detect pesticide-induced Raman spectral changes (Hernandez-Castillo et al., 2015); and colorimetric detection based on color changes in calixarene-functionalized silver nanoparticles (Xiong and Li, 2008). Overall, the non-fluorescence-based systems for monitoring pesticide interactions with calixarenes, while effective in detecting pesticides, suffer from challenges in implementation in complex, real-world environments.

Overall, calixarenes tend to provide good opportunities for selective binding and detection of pesticides, yet suffer from disadvantages that include the lack of a built-in transducing (i.e., signaling) element (Schneider, 2019). While this disadvantage is also apparent for cyclodextrins and cucurbiturils (detailed above), there are virtually no literature-reported examples in which fluorescent moieties are attached directly to the calixarene core for fluorescence-based pesticide detection. In contrast, numerous examples of such cases for cyclodextrin and cucurbituril-based sensors have been reported (*vide supra*). Nonetheless, advantages to calixarenes include relatively straightforward synthetic access (Wang, 2012), as well as the ability to attach calixarenes to a variety of other structures, (i.e., nanoparticles (Uttam et al., 2020) and gold surfaces ((Garnier et al., 2020), *vide supra*) to obtain hybrid pesticide sensors. Binding affinities for analytes inside calixarene hosts are relatively similar to those measured for cyclodextrin hosts, both of which are noticeably weaker than observed binding affinities inside cucurbituril.

#### Pillararenes

Pillararenes are a relatively novel class of synthetic macrocycles, first reported by Ogoshi and co-workers in 2008 (Ogoshi et al., 2008). They are similar to calixarenes, but the linkages between the phenols lead to pillar-like, highly symmetrical structures (Behera et al., 2020). Pillararenes have been used in a variety of supramolecular applications, including for the binding and removal of organic toxicants from contaminated aqueous environments (Fernando et al., 2019); as components of solid-state crystalline structures (Li et al., 2020a); and as nanochannels for biomimetic research (Sun et al., 2020). Like the macrocycle classes previously discussed, pillararenes rely primarily on hydrophobic association to bind small-molecule hydrophobic guests (Shen et al., 2018), with particular examples reported using electrostatic interactions (Liu et al., 2019b) and/or intermolecular hydrogen bonding (Han et al., 2020a) as co-occurring mechanisms.

**FIGURE 8:**
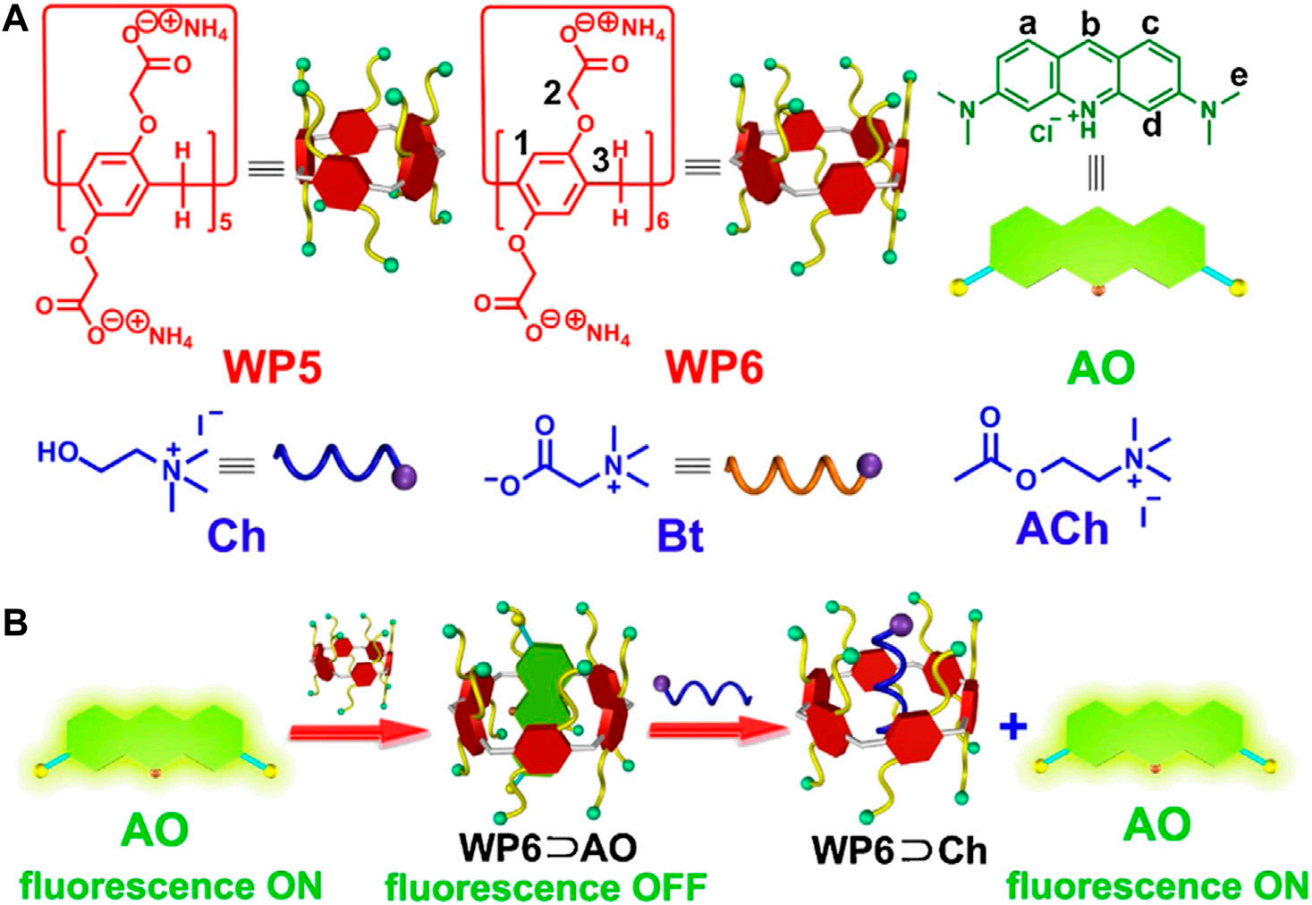
**(A)** Structures of the components used for the pillarene-based choline sensor **(B)** Schematic illustration of how displacement of the acridine orange moiety leads to a turn-on fluorescence sensor for choline. Reprinted from reference Hua et al. (2018).

The use of pillararenes for fluorescence-based pesticide detection has been relatively underdeveloped to date. In one example, a highly electron-rich anionic pillararene bound acridine orange with high affinity, resulting in quenching of the acridine orange emission (Figure 8) (Hua et al., 2018). Subsequent introduction of choline resulted in a full restoration of the acridine orange color and fluorescence emission. While not technically a pesticide, choline has found important use in detection systems as an indicator of pesticide-induced disruption (Vivek and Jain, 2020); as such, its detection using fluorescence-based methods has relevance to this review.

In another example, Zhang et al. reported that coumarin moieties could be directly linked to a pillar [5]arene (Zhang et al., 2015), resulting in a structure that bound methyl parathion with high affinity (2.38 × 10^−4^ M^−1^) leading to fluorescence quenching. Although good selectivity was observed for methyl parathion, no explanation for the basis of such selectivity was provided, nor was an explanation provided for how the addition of methyl parathion induced such strong quenching.

Finally, a variety of researchers have reported that pillararenes bind paraquat with high affinities, resulting in binding-induced fluorescence changes. In one example, a covalently linked tri-pillar[5]arene responded to the binding of paraquat with a near-complete fluorescence quenching (Wang et al., 2021). Moreover, tri-pillar[5]arene was more efficient at removing paraquat from solution compared to the monomeric pillar[5]arene, indicating cooperativity. Finally, a comparison between laboratory-grade paraquat and the commercially available paraquat insecticide indicated even higher efficacy in removing the commercial pesticide from contaminated environments. Of note, adsorbing pillar [5]arene on a graphene support allowed it to retain its ability to bind paraquat (Tan et al., 2019), including in the complex environments of living cells (Mao et al., 2016).

**FIGURE 9:**
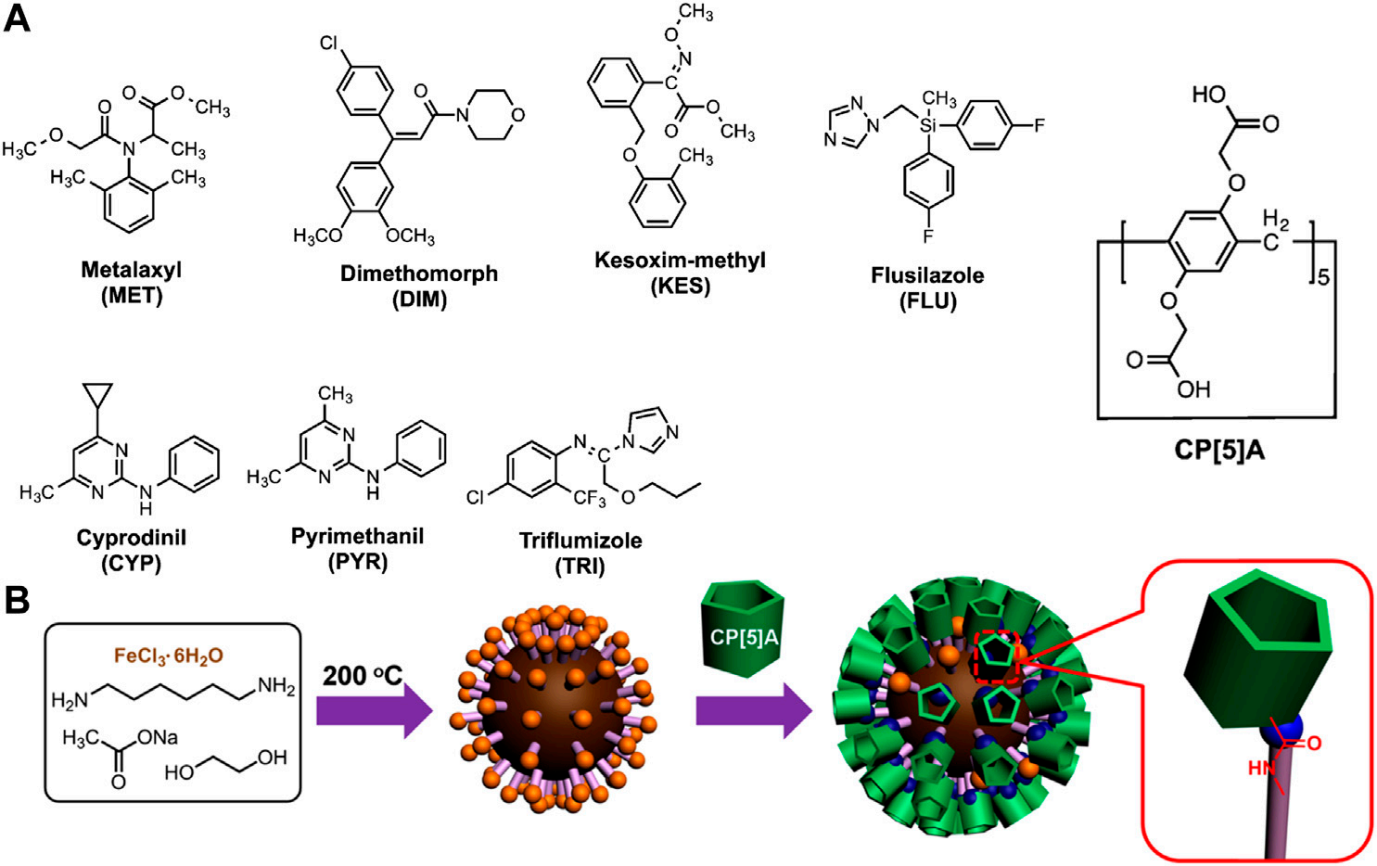
**(A)** Structure of carboxylatopillar [5]arene CP [5]A and pesticides investigated **(B)** Schematic illustration of the synthesis of CP [5]A-iron oxide nanoparticles and their removal using magnetic probes. Reprinted from reference Tian et al. (2013).

Other examples have reported the detection of pesticides with pillararenes using non-fluorescence-based methods. In one example, carboxylatopillar [5]arene (CP [5]A, Figure 9A) was used to bind a variety of pesticides (Tian et al., 2013). Because the host was immobilized on the surface of magnetic nanoparticles, pesticide binding was followed by effective magnetic-based pesticide removal (Figure 9B). In another example, electrochemical-based detection of the binding of pesticides to a pillararenes was reported with extremely high sensitivity (Shamagsumova et al., 2015; Hou et al., 2019).

Compared to calixarenes, a notable advantage of pillararenes includes the fact that the more rigid and symmetrical macrocyclic hosts can often bind small molecule guests with higher binding affinities (Liu et al., 2019b). Nonetheless, pillararene syntheses are generally more complex than those of analogous calixarenes (Guo et al., 2018), and often lead to mixtures of isomers that can be difficult to purify. Additionally, the high stability of pillararene architectures means that they have been used effectively to accomplish environmental remediation of contaminated aqueous solutions (Fernando et al., 2019), and they have also been used effectively when adsorbed (covalently or non-covalently) (Yang et al., 2014) to solid supports (Lou and Yang, 2020).

#### Cyclophanes and Synthetic Macrocycles

Cyclophanes, defined as hydrocarbon macrocycles with aromatic units bridged by non-aromatic units, were first reported in 1964 (Longone and Simanyi, 1964). While other synthetic approaches to a broad variety of cyclophane isomers followed quickly (Sato et al., 1966; Fujimoto et al., 1967; Reich and Cram, 1967), the utility of such structures as supramolecular hosts was relatively slow to develop. Pioneering work in this area was done by Francois Diederich and co-workers, who used cyclophanes as effective supramolecular catalysts (Diederich et al., 1988; Diederich and Lutter, 1989). Later, Stoddart and co-workers built on this initial work to use cyclophanes for supramolecular devices (Asakawa et al., 1999) and complex, stimuli-responsive architectures (Zhou, et al., 2019; Ashton et al., 1999). Nonetheless, cyclophanes as hosts for pesticide detection have rarely been reported, and their use as fluorescence-based pesticide sensors is virtually unknown.

Nonetheless, other synthetic macrocycles have been used effectively for pesticide detection. In one example, a highly fluorescent tetraphenylene boronate macrocycle that self-assembled into nanoparticles was used to bind 2,6-dichloro-4-nitroaniline, resulting in rapid fluorescence quenching and a 2 ppm detection limit (Hoshi et al., 2018). The mechanism by which such quenching was achieved was attributed to both to the quenching efficacy of nitroaniline, as well as the fact that the introduction of the analyte changed the nanoparticle morphology. Notable drawbacks to this report include the fact that selectivity for this analyte compared to other structurally similar compounds was not investigated, nor was the application of the system to real-world environments reported.

Synthetic macrocycles have significant advantages, including the ability to rationally design a structure for targeted binding of particular pesticide analytes (Ferracane et al., 2020). Drawbacks to such macrocycles include the general need for complex, multi-step syntheses, which are often plagued by low yields, particularly in the cyclization step (Mortensen et al., 2019). While efforts to improve the yield of synthetic macrocycle cyclizations, including the use of templation (Barnes et al., 2013) and effective high dilution conditions (Choo et al., 2020) have partially addressed this issue, challenges still remain. Moreover, such macrocycles are often less effective in providing a completely enclosed environment for the target analytes compared to the other architectures (cyclodextrins, cucurbiturils, etc.) detailed above, which in turn can result in lower binding affinities, sensitivities, and selectivities for target pesticide analytes.

### Fluorescent Macrocycles

Although fluorescent macrocycles have been extensively reported in the literature (Tamgho et al., 2017; Feng et al., 2018; Yang et al., 2020; Yang and Jiang, 2020), they have rarely been used directly for fluorescence-based pesticide detection. Most cases of fluorescent supramolecular architectures used for pesticide detection are those in which a fluorescent moiety is attached to a non-fluorescent macrocycle (*vide supra*) (Champagne et al., 2019). Another category of relevant macrocycles are those in which two or more macrocycles are covalently linked *via* a fluorescence linker, thus providing a built-in fluorescence signal for analyte binding. In one such example, the use of 3,3′-benzidine as a covalent linker between two cyclodextrins led to a fluorescence sensor for the pesticide dithianon (Tian et al., 2012). In another example, a trihydroxy-phenol core was covalently linked to three β-cyclodextrins (Mallard-Favier et al., 2009). The resulting construct displayed strong fluorescence in the absence of pesticides that was quenched with the addition of pendimethalin, leading to detection limits in the low micromolar range (0.8–4 µM). While highly sensitive, the selectivity of this system for binding pendimethalin in the presence of other small molecules was not explored.

**FIGURE 10:**
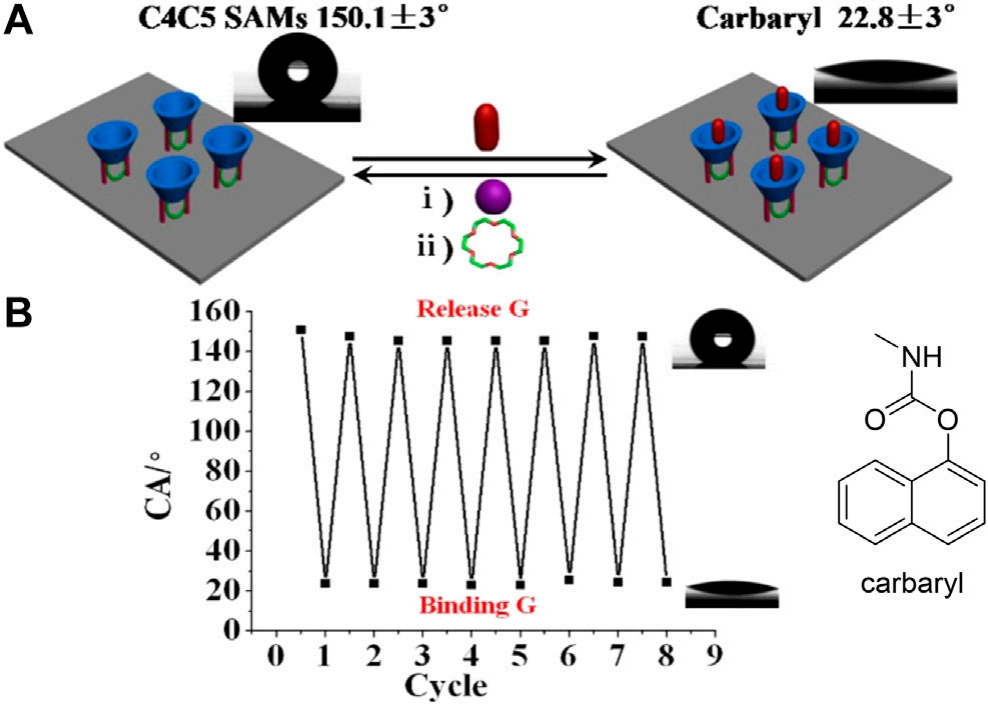
**(A)** Schematic illustration of how binding of carbaryl (abbreviated as “G”) causes changes in the calixarene-containing monolayer and how such binding can be reversed with the addition of potassium cations **(B)** Measurements of the reversibility of the system through monitoring changes in the contact angle (CA) as a function of cycle number. Reprinted from reference Luo et al. (2015).

Moreover, the use of covalently attached fluorescent moieties includes attachment to non-cyclodextrin scaffolds. In one example, Yilmaz et al. reported that covalently linking 1,8-naphthalimide fluorophores to calix[4]arenes led to structures that displayed notable decreases in their fluorescence emission upon binding pesticides (Yilmaz et al., 2018). Interestingly, the addition of urea to the supramolecular complex led to a triggered release of the encapsulated pesticide guest, providing good system reversibility. Controlled release from a functionalized calixarene was also seen in the case of a calixarene covalently linked to a crown ether; binding of potassium cations to the crown ether resulted in conformational changes that released the bound pesticide (carbaryl) from the main calixarene cavity (Figure 10) (Luo et al., 2015b).

Of note, one clear advantage to the use of fluorescent macrocycles is the fact that signaling elements are built right into the structure of the macrocycle, which provides close contact between that element and the pesticide analyte that binds in the macrocycle cavity (Jiang et al., 2020c). Although the potential for such close contacts to lead to improved sensor performance exists, in many cases the sensitivity and selectivity obtained from using fluorescent macrocycle sensors are essentially indistinguishable from those obtained using other macrocycle scaffolds (Sethupathi et al., 2020). Additional advantages of such macrocycles include the ability to attach them to solid supports, including paper (Kumar et al., 2018) and glass backing (Wienke et al., 1996), for the development of solid-state sensors (Lvova et al., 2018).

## Nanomaterial-Based Pesticide Detection

Nanomaterials, broadly defined as structures with nanoscale dimensions (Joachim, 2005), encompass a range of structures, including quantum dots, nanocrystals, and nanoparticles. Unique properties of nanomaterials are due to their high surface-to-volume ratio (Mukhopadhyay et al., 2005), which means that surface structure has a disproportionate effect on their overall properties (Kumar et al., 2020b). Nanomaterials have seen extensive use in organic optoelectronics (Pron et al., 2019); in battery (Pan et al., 2020) and battery-electrolyte research (Glynos et al., 2020); in medicine (Khan et al., 2020) and targeted therapies (Wang et al., 2020a), and in the development of novel chemical sensors (Torrinha et al., 2020).

The use of nanomaterials for pesticide detection is well-precedented, with nanomaterials responding to the presence of pesticides *via* changes in their electrochemical, colorimetric, and Raman spectroscopic signals (Li et al., 2019). In addition, pesticide-induced fluorescence changes in nanomaterials have also been reported. This section will review these nanomaterial-based fluorescence pesticide sensors, with the section sub-divided based on nanomaterial type. Of note, other review articles that discuss nanoparticle-based pesticide detection have been published, and the interested reader is directed there for more information (Kumar et al., 2015; Bapat et al., 2016; Farka et al., 2017; Krishnan et al., 2019; Kolataj et al., 2020).

### Quantum Dots

**FIGURE 11:**
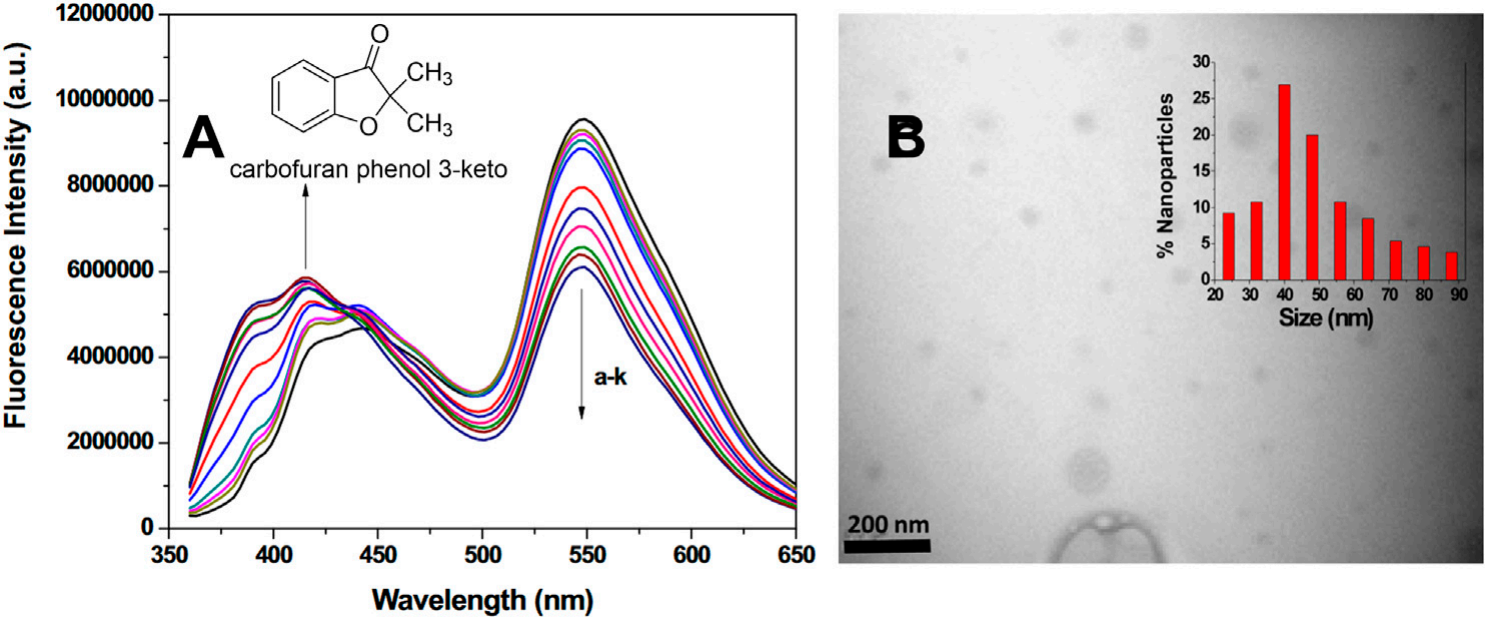
**(A)** Fluorescence changes of carbon dots coated with vitamin B12 with the introduction of the pesticide derivative carbofuran phenol 3-keto **(B)** TEM images of the carbon dots with an average size distribution shown in the inset. Reprinted from reference Campos et al. (2017).

Quantum dots are defined as highly luminescent materials up to a few nanometers in size (Kash, 1990). These materials have been used extensively in chemical sensors (Ganesan and Nagaraaj, 2020), due to their high sensitivity to the local environment (Kim et al., 2018) and straightforward modification procedures (Mehta et al., 2019; Li et al., 2015). In one example, carbon quantum dots coated with vitamin B12 exhibited dual fluorescence emission from excitation at 355 nm: emission at 417 nm due to the carbon dots, and emission at 550 nm due to energy transfer from the carbon dots to the vitamin B12 coating (Figure 11) (Campos et al., 2017). Introduction of the pesticide carbofuran resulted in a disruption of the energy transfer, leading to a decrease in the fluorescence emission at 550 nm and an increase in the fluorescence emission observed at 355 nm. This dual-emission system was used as an effective ratiometric sensor for carbofuran, with the authors hypothesizing that carbofuran forms a charge transfer complex on the surface of the quantum dot, which leads to static quenching of the carbon dot emission and a shutting down of the energy transfer pathway. Good selectivity compared to other pesticide analytes was demonstrated, as was the ability to detect carbofuran in complex, real-world matrices.

Another example using carbon quantum dots was reported by Hou et al., in which iron (III) cations were used as fluorescence quenchers (Hou et al., 2020). Subsequent introduction of glyphosate resulted in strong interactions between the glyphosate and the iron (III) cations, which removed the cations from the proximity of the carbon dots and restored the fluorescence. Experimental work indicated that the mechanism of fluorescence quenching between the carbon dots and iron cations occurred *via* electron transfer. Moreover, although good sensitivity for glyphosate was reported, the degree of selectivity was not. Based on the system construct, it is likely that other pesticides that bind iron cations may induce similar fluorescence responses. Other carbon dot-based fluorescence sensors have also been reported (Bera and Mohapatra, 2020).

In general, quantum dots are intriguing structures as sensor materials, with key advantages coming from their extremely small size and the ability to rationally tune their photophysical properties *via* size modification of the dots (Huang et al., 2019). In fact, a recently published report indicated that molecular imprinted quantum dots can be used to detect the pesticide propanil in complex, real-world environments (Liu et al., 2020), and another report showcased the general applicability of emissive carbon dots in detecting analytes with high relevance to pesticides (Li et al., 2018b). Nonetheless, the limited toolbox available for significant structural modification means that most quantum dot-based sensors rely on the use of quantum dots in combination with other nanomaterials, with the resulting hybrid structures demonstrating high utility in a range of environments. The interested reader is directed to Section v (“Nanomaterials containing more than one nanostructure”) for more information about these architectures.

### Nanocrystals

Nanocrystals, defined as crystals with nanoscale dimensions, are a subcategory of nanomaterials with well-defined crystal structures (Gleiter, 1992). They have been used extensively in applications including optoelectronics (Zhou et al., 2020a) and materials science (Gemmi et al., 2019), and their use in chemical sensors has been reported (George et al., 2020). One example of nanocrystal-based pesticide sensors was reported by Dey and Das in 2019, using cadmium sulfide nanocrystals as energy donors with Eosin Y as an energy acceptor, with excitation of the nanocrystals resulting in energy transfer to and emission from the Eosin Y fluorophore (Dey and Das, 2019). Extensive work confirms that this energy transfer occurs *via* Förster Resonance Energy Transfer (FRET), based on the significant spectral overlap between the donor emission and acceptor absorption spectra and the strong distance dependence observed (Scholes, 2003). The addition of chlorpyrifos (Lasagna et al., 2020) to the system resulted in an increase in the fluorescence emission of the nanocrystals due to pesticide-induced aggregation. The energy transfer peak observed as Eosin Y dye emission, however, remained unaffected. Finally, the system was able to detect chlorpyrifos at ppb-level concentrations.

### Nanoparticles

The vast majority of nanomaterial-based fluorescent pesticide sensors use nanoparticles, defined as spherical particles with nanometer-scale dimensions. These nanoparticles can be made of metals (i.e. cadmium, zinc, etc.) (Zhou et al., 2020b); semi-metals, (i.e. silicon, germanium, etc.); or non-metals, (i.e. organic polymer-derived nanoparticles). Each type of nanoparticle will be discussed in turn.

**FIGURE 12:**
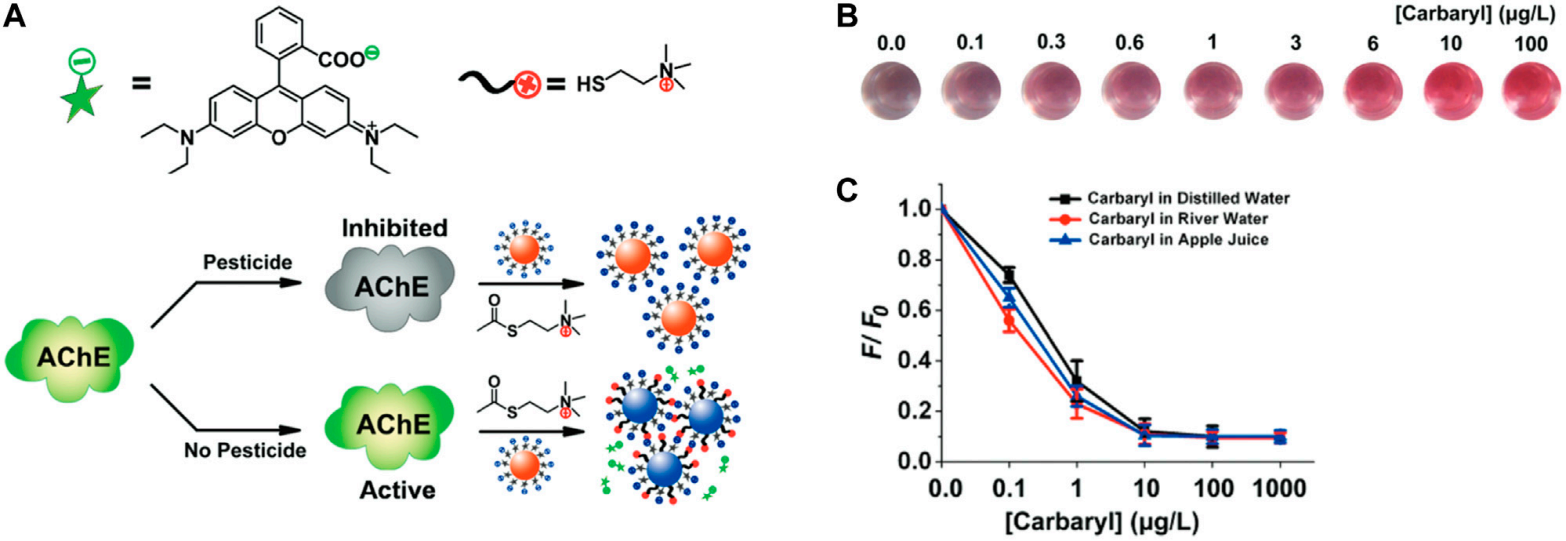
**(A)** Schematic illustration of how carbaryl detection can be accomplished based on inhibition of the activity of acetylcholinesterase (AChE) **(B)** Color change with increasing concentrations of carbaryl (left to right) **(C)** Changes in the fluorescence ratio (*F*/*F*_0_) with increasing concentrations of carbaryl in distilled water (black line), river water (red line), and apple juice (blue line). Reprinted from reference Liu et al. (2012).

One example using metallic nanoparticles for pesticide detection used gold nanoparticles with rhodamine B to detect organophosphorous and carbamate pesticides (Figure 12) (Liu et al., 2012). In the absence of pesticides, active acetylcholinesterase (AChE) leads to the production of thiocholine, which binds to the rhodamine on the nanoparticle surface and facilitates the formation of aggregates with unique fluorescence emission. Introduction of pesticides that are known AChE inhibitors blocks the formation of thiocholine and the concomitant nanoparticle aggregation. As a result, monomeric nanoparticles are formed with a different fluorescent profile, enabling a dual-readout pesticide sensor. Other examples of sensors based on AChE activity affecting the fluorescence emission of metallic nanoparticles have also been reported (Long et al., 2015; Luo et al., 2017).

A second example using gold nanoparticles for fluorescence pesticide sensing was reported by Su et al., in which the pesticide cyromazine (Mansoor and Shad, 2020) selectively interacted with the gallic acid-containing nanoparticle coating (Su et al., 2012). These interactions destabilized the nanoparticles and led to a marked fluorescence decrease that coincided with the formation of non-fluorescent aggregates. Of note, strong selectivity for cyromazine was observed compared to other pesticide analytes and other potential interfering anions and cations, and the ability of the system to detect cyromazine in the complex matrix of a milk sample was also demonstrated.

An intriguing example using metallic nanoparticles was reported by Walia and Acharya, in which fluorescent cadmium sulfide nanoparticles were used for the detection of organochlorine pesticides (Walia and Acharya, 2014). This sensor operated through pesticide-induced increases in the fluorescence emission of the cadmium sulfide nanoparticles. These changes, of note, were specific to the organochlorine pesticides, with other pesticide classes leading to no measurable fluorescence changes. The authors explain that pesticide-induced nanoparticle aggregation is responsible for the observed fluorescence changes, with the amine and carboxylic acid functionalities of the glutathione coating facilitating productive pesticide-nanoparticle interactions. While the selectivity of this system between classes of pesticides was good, the selectivity within the pesticide class, (i.e. between different organochlorine pesticides) was not addressed.

Metallic nanoparticles were also able to detect pesticides *via* fluorescence energy transfer. Such systems were particularly effective when the donor contained a lanthanide that could be excited in the near-infrared spectral region, absorb multiple photons, and emit visible light. This process, called “upconversion,” was used by Long et al. in nanoparticles that contained europium and ytterbium energy donors combined with gold nanoparticle energy acceptors (Long et al., 2015). Upconversion enabled excitation of the energy donors to occur *via* laser excitation, resulting in emission from the gold nanoparticles that was quenched in the presence of the pesticide analyte, parathion methyl. Of note, excellent selectivity for this pesticide was demonstrated compared to other co-existing analytes, and the ability to detect parathion methyl in complex samples was demonstrated. Other examples of the use of upconversion nanoparticles for fluorescent pesticide detection have also been reported (Hu et al., 2016; Yang et al., 2019; Yu et al., 2019; Li et al., 2020b).

Semi-metal nanoparticles have also been reported for fluorescence pesticide detection. The boronate-containing macrocycles discussed in the macrocycle section (*vide supra*) constitute one such example (Hoshi et al., 2018). ^308^ These structures form nanoparticles with strong fluorescence emission (31% relative quantum yield) in the yellow-green spectral region that respond to the presence of 2,6-dichloro-4-nitroaniline with a strong decrease in the observed fluorescence intensity.

Finally, metal-free nanoparticles that respond to the presence of pesticides with measurable fluorescence changes have also been reported. In one example, a poly-fluorene-poly-benzothiadiazole was fabricated into nanoparticles using nanoprecipitation methodology (Jiang and McNeill, 2017). These fluorescent nanoparticles were used as energy donors to manganese oxide nanosheet acceptors, resulting in fluorescence quenching. Introduction of pesticides to this construct disrupted the energy transfer, which in turn led to restoration of the innate nanoparticle fluorescence. Energy transfer involving fluorescent organic nanoparticles has been implicated in a variety of sensor schemes (Y. Wang et al., 2020; Kayhomayun et al., 2020; Abdolmohammad-Zadeh et al., 2021), but has been used only rarely in pesticide detection to date.

### Other Nanostructures

In addition to the aforementioned nanostructures, there are a variety of other shapes and structures that have been used for pesticide detection. In one example, europium-doped titanium oxide nano-powder responded to the presence of chlorpyrifos with significant fluorescence quenching (Yao et al., 2011). Computational and experimental work indicated the existence of an electron transfer mechanism, and the limit of detection using this system was determined to be in line with or better than other reported chlorpyrifos sensors (Wang et al., 2020f; Xu et al., 2020).

Nanocrystals, nanoparticles, and other nanostructures each have unique advantages and drawbacks to their use as fluorescent sensor materials. Nanoparticles are generally the easiest structure to access synthetically (Tarannum et al., 2019), and therefore enjoy practical advantages that nanocrystals and other nanostructures do not. Nonetheless, nanocrystals enjoy extremely high sensitivity to a range of analytes due to their precise crystalline structure (Tang et al., 2020a), and other nanostructures can have tailored aspect ratios to facilitate similarly highly sensitive responses (He et al., 2020b). Notable drawbacks to the use of nanomaterials include the fact that they usually contain toxic metals; this toxicity is a concern throughout the life cycle of the nanomaterial and can lead to complicated disposal procedures (Domi et al., 2020).

### Nanomaterials Containing More than One Nanostructure

Oftentimes, more than one type of nanostructure is combined to yield a high performing sensor (Yan et al., 2018b). In one example, researchers used the fact that gold nanoparticles effectively quench the fluorescence of CdTe quantum dots *via* an inner filter effect to design an effective pesticide sensor (Guo et al., 2013). In this system, the presence of acetylcholinesterase (AChE) led to the formation of thiocholine, which caused nanoparticle aggregation, a reduction of the inner filter effect, and a concomitant fluorescence increase. The presence of carbamate pesticides, known inhibitors of AChE, shut down thiocholine production, preventing nanoparticle aggregation and quenching the system fluorescence. Of note, other systems that rely on the inner filter effect to mediate interactions between metallic nanoparticles and quantum dots have also been reported (Yan et al., 2014; Yan et al., 2015; Guo et al., 2016).

In a related example, Li et al. reported that silver nanoparticles quenched the fluorescence emission of graphene quantum dots (Li et al., 2020d). In the absence of additional influence, therefore, no fluorescence is observed. However, introduction of AChE and the concomitant production of thiocholine from acetylcholine leads to binding of the thiocholine to the silver nanoparticles, removing them from the proximity of the quantum dots and restoring the fluorescence. Parathion methyl, a known inhibitor of AChE (Talley et al., 2020), disrupts the formation of thiocholine and restores the fluorescence quenching. The system worked well to detect parathion methyl in real-world samples, although the selectivity for parathion methyl compared to other pesticides that are known AChE inhibitors was not reported. Other researchers used B,N-doped carbon quantum dots as fluorophores and gold nanoparticles as fluorescence quenchers to develop essentially the same pesticide detection scheme for the detection of carbaryl (Chen et al., 2020b). Other related examples have also been reported (Wu et al., 2017b; Yang et al., 2018).

Another example was reported in which energy transfer between ytterbium-containing nanocrystals and gold nanoparticles led to pesticide detection (Wang et al., 2013). This system, in the absence of pesticides, displays strong luminescence due to the presence of ytterbium. Introduction of the pesticide analyte, cartap, to the system results in aggregation of the nanocrystals and concomitant energy transfer from the aggregated nanocrystals to the gold nanoparticles. Such energy transfer, in turn, reduces the ytterbium-based luminescence and leads to pesticide-induced fluorescence quenching. In an additional example using gold nanoparticle-involved energy transfer, energy transfer between gold nanoparticles and graphitic carbon nitride was disrupted by the presence of pesticides acting as AChE inhibitors to enable effective detection (Xie et al., 2018).

## Metal-Organic Framework Pesticide Detection

Metal-organic frameworks (MOFs) were first reported by Yaghi and co-workers in 1995, when they reported that a microporous metal-organic framework with high rigidity facilitated the binding of small molecule guests within the MOF cavities (Yaghi et al., 1995). More broadly, these structures are defined as a highly porous three-dimensional network of metal cations connected by organic ligands (Furukawa et al., 2013). Applications of MOFs are extensively reported in the literature, and include environmental remediation *via* pollutant binding inside the MOF structure (Ejeromedoghene et al., 2020); supramolecular catalysis by encapsulating reactants (Ghasemzadeh et al., 2020); controlled crystallization *via* topological effects of MOF incorporation (Wang et al., 2020i), and a variety of other applications (Jones et al., 2018; Ding et al., 2020; Heo et al., 2020).

Pesticide binding in MOFs was first reported in 2010, when pesticides were removed from lettuce *via* solid-phase extraction using a lanthanide-based MOF (Barreto et al., 2010). While the recovery of doped pesticides was very high (78–107%), identification and quantification of the pesticides was done using GC-MS, which added significant time and cost to the process. In the same year, researchers reported that a biphenyl-containing MOF could bind low concentrations of organophosphate pesticides, with the success of such binding measured using electrochemical analysis (Wen et al., 2010). A variety of other electrochemical-based detections of pesticide binding in MOFs have since been reported (Nagabooshanam et al., 2019; Tu et al., 2019; Chansi et al., 2020).

**FIGURE 13:**
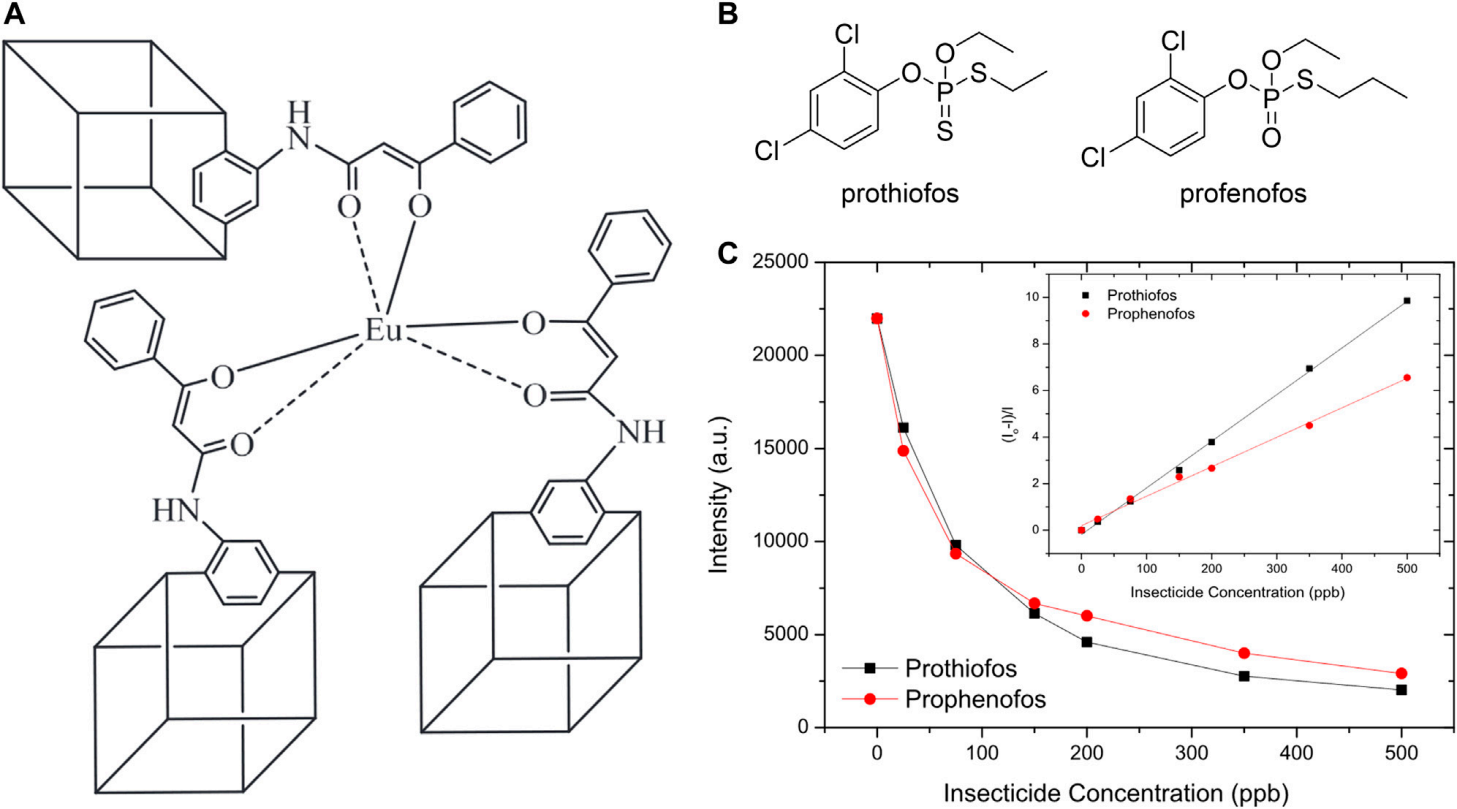
**(A)** Schematic illustration of the europium-complexed MOF used for pesticide detection herein **(B)** Structures of the two pesticides, prothiofos and profenofos, investigated herein **(C)** Illustration of the decrease in fluorescence emission observed with increasing concentrations of prothiofos and prophenofos. Inset shows a linear plot of the concentration of the insecticide *vs*. (*I*_o_-*I*)/*I*. Reprinted from reference Abdelhameed et al. (2020).

In addition to the significant reports of electrochemical-based detection using MOFs, several recent examples (2019–2020) indicate that pesticide binding in MOFs can be measured using fluorescence changes. In one example, IRMOF-3, a commonly used, zinc-containing MOF (Sagara et al., 2005; Braga and Longo, 2005), was covalently modified post-fabrication to add europium-targeting ligands (Figure 13) (Abdelhameed et al., 2020). Once mixed with europium, the resulting supramolecular structure displayed strong fluorescence emission in the near-infrared region (Kang et al., 2020), with marked decreases in emission observed after the addition of pesticides prothiofos and profenofos (Mortensen, 2006). Computational methods were invoked to explain the favorable pesticide-MOF interactions, as well as to explain the selectivity for these guests. Particular advantages to this method include the versatility of post-synthetic modification of MOFs.

In another example, a zinc-containing, water-soluble MOF was used to bind a variety of common contaminants, including heavy metals, anions, and 2,6-dichloro-4-nitroamine, a nitroaromatic compound with insecticidal activity (Xu et al., 2019c). All of the guests led to quenching of the MOF’s fluorescence emission, an effect that was attributed to proximity-induced fluorescence resonance energy transfer (Wang et al., 2019b). Because such energy transfer requires spectral overlap between the emission spectrum of the energy donor and the absorption spectrum of the acceptor (*vide supra*), high levels of selectivity were observed (i.e. only those that demonstrate sufficient spectral overlap were detectable).

A different approach was reported by Zhang and co-workers, who fabricated a supramolecular complex of a MOF host with a rhodamine guest that displayed solid-state fluorescence energy transfer from the MOF to the rhodamine (Bera and Mohapatra, 2020). In the absence of any pesticide, fluorescence emission occurred from both the MOF ligands (at 363 nm) and the rhodamine (at 580 nm) *via* MOF excitation. Introduction of parathion-derived pesticides resulted in fluorescence quenching of both emission signals, with the ratio of quenching of the signals highly dependent on the pesticide. Computational work attributed the mechanism of such analyte-induced changes to a combination of an inner filter effect and resonance energy transfer.

Detailed computational analysis of luminescent MOFs used for pesticide detection was performed by Halder and co-workers, who analyzed a copper-containing MOF with bridging 4,4′-bipyridine ligands and succinate dianions (Halder et al., 2019). Density functional theory revealed that the oxygen moieties of succinate were instrumental in coordinating to NH and OH groups of the pesticide guests, with additional benefit derived from aromatic π-π stacking between the bipyridine ligands and aromatic pesticides. Moreover, a direct comparison with a cadmium MOF revealed that the copper component provided significant benefit, with excellent selectivity towards atrazine- and dicofol-derived pesticides.

An intriguing combination of two supramolecular classes was reported by Yu et al., who used two-dimensional MOF nanosheets with calix[4]arenes to detect pesticides (Yu et al., 2020). These nanosheets were formed by fabricating a three-dimensional MOF (based on cadmium metal nodes and calixarene-containing bridging ligands), followed by exfoliation to form the nanosheets. Introduction of glyphosate resulted in a substantial increase (up to 2.4-fold) of the fluorescence emission, attributed to glyphosate-induced rigidification of the system that facilitated the electron transfer required for the observed fluorescence emission. Additional information in support of pesticide-induced rigidification was provided, including temperature experiments, wherein low temperatures caused similar rigidification and analogous fluorescence changes.

Finally, reusability of luminescent MOF sensors was demonstrated by a number of research groups. In one example, a europium-containing luminescent MOF was used to detect pesticide metabolites in urine samples, with the presence of those analytes leading to fluorescence quenching (Wang et al., 2020b). Washing of the MOF with deionized water was sufficient to regenerate the sensor, leading to no decrease in performance after three cycles. While this report provided a good proof of concept, only a limited number of washing cycles was reported. In a second example, a magnetic covalent organic framework (COF) was used to detect atrazine, chlorpyrifos, and diquat dibromide in contaminated aqueous solutions (Romero et al., 2020). Pesticide binding to the COF was followed by magnetic solid-phase extraction to remove the pesticides, and regeneration up to five cycles was accomplished. While this system showed remarkable pesticide removal capabilities, the number of regeneration cycles was still limited.

Known advantages of MOFs include the fact that they are extremely easy to access synthetically: merely by mixing the metal node and chelating ligand, one can access a broad range of structures (Zhao et al., 2020). Once formed, the MOFs can bind a range of structures in their highly porous interiors, leading to the ability to bind and detect a broad range of pesticides (Karmakar et al., 2017). Moreover, this binding ability can be exploited not only for high affinity detection but also for simultaneous detection and environmental remediation (Lu et al., 2021). Disadvantages of MOFs include the fact that they can easily decompose to their constituent metal and ligand components, which precludes their use in a broad range of complex environments (Wen et al., 2020). Moreover, although the synthetic access to the MOFs themselves is straightforward, the ability to execute synthetic modifications on the MOF after its fabrication remains elusive (Al Amery et al., 2020). Finally, the cavity sizes in the highly porous MOF structure often bind a range of analytes, with selectivity for a particular analyte of interest only moderate.

## Pesticide Detection in Different Phases

Significant differences in pesticide detection can occur if pesticide detection operates in the solid-state, in solution, or in the vapor phase. Each of these phases are discussed in turn.

### Solid-State Pesticide Detection

The detection of pesticides in solid-state samples occurs most commonly for produce, plants, and soils. One mechanism of pesticide detection in vegetables was reported by Schechter and co-workers (Hassoon and Schechter, 2000), who sprayed an eggplant peel with a solution of Nile Red, a fluorophore that reacts to the presence of methoxychlor with notable fluorescence changes (Hassoon and Schechter, 1998). In this report, eggplant peel that had been doped with the methoxychlor pesticide displayed different solid-state fluorescence properties than eggplant peel lacking such pesticides. While good (nanogram-level) selectivity was achieved, broad-based applicability of the system for other vegetables and for other pesticides was not demonstrated.

A more generally applicable fluorescence sensor for contaminated produce was recently reported by Tang et al., in which multicolor nitrogen dots (M-Ndots) were used for the detection of thiram and chlorpyrifos (Tang et al., 2020b). These M-Ndots were formed from 5-amino-*1H*-tetrazone and *p*-phenylenediamine, leading to fluorescent structures that were embedded into a gas membrane separation device. Combining the multicolor nitrogen dots with copper and iron allowed them to detect thiram and chlorpyrifos, respectively, based on the pesticides’ affinity for the metal cations. Interestingly, effective detection in lychee, pear, orange, lettuce, and cucumber samples was also observed, with the new method validated by comparison to an LC-MS assay.

Effective pesticide detection in plants is even more critical, as understanding pesticide contamination at this stage can affect agricultural decisions. In one example, researchers found that quantum dots were effective in detecting carbamate pesticides in medicinal plants (Wei et al., 2018). In this system, AChE-generated hydrogen peroxide quenched the quantum dot fluorescence. In the presence of carbamate pesticides that were effective AChE inhibitors, hydrogen peroxide was not produced and the quantum dot fluorescence signal remained strong. This system operated effectively to detect carbamate pesticides in contaminated medicinal plants, with limits of detection for carbofuran (5 nM) lower than maximum regulatory limits (Vidal et al., 2007).

In another example of solid-state pesticide detection, the detection of glyphosate in soil samples was accomplished using carbon dot-labeled labeled magnetic beads (Wang et al., 2016a). In this sensor, antibodies for glyphosate were attached to fluorescent carbon dots. After binding between the glyphosate and its designed antibody enabled fluorescence-based detection, the use of the corresponding antigen attached to magnetic iron oxide nanoparticles allowed for the straightforward removal of the glyphosate from the contaminated samples with limited interference from other biological analytes. Recovery percentages recorded were high (>75%), with low detection limits and high levels of selectivity reported as well.

### Solution-State Pesticide Detection

The most common way to detect pesticides is in the solution-state, particularly in aqueous environments. Doing so provides limited interference from other substances and enables straightforward analysis. Moreover, monitoring the pesticides that enter water supplies provides a rapid way to understand overall pesticide contamination, ecosystem health, and the public health risk that results from pesticide exposure. The majority of work mentioned in other sections of this article focuses on pesticide detection in solution, and a few additional examples are added here.

In one example, an on-site early warning system for pesticide contamination was established, using the ability of UV irradiation to cause controlled pesticide degradation to fluorescent photoproducts (Bakhoum et al., 2020a). Because the degradation products were dependent on the structure of the starting pesticide, good levels of selectivity were observed in identifying the pesticide contaminant. This method operated effectively in real-world water samples to detect contamination. Nonetheless, the requirement for high energy UV irradiation to initiate the photodegradation process can detract from the practical applicability of this approach.

A second example was reported by Wilkommen et al., who used a fluorescence-based pesticide sensor to detect pesticides both in contaminated soil and in contaminated shallow groundwater (Willkommen et al., 2021). The pesticide of interest, flufenacet, has been found in a number of groundwater locations (Tauchnitz et al., 2020) and has significant suspected toxicity (Bunzel et al., 2015). Of note, the general applicability of this method to multiple phases allowed the researchers to track the fate of the pesticide analyte as well as of its most common degradation byproduct, flufenacet ESA. Other detection methods that operate for both soil and aqueous detection have also been reported (Bakhoum et al., 2020b; Hong et al., 2020).

In one final example of solution-state detection, researchers reported the use of a solid-state paper sensors to accomplish effective aqueous detection (Wang et al., 2020h). In this example, a triphenylamine-modified polysiloxane hybrid structure responded to the presence of 4-nitrophenol with a strong fluorescence quenching due to electron transfer from the electron rich triphenylamine to the electron deficient nitrophenol. This system worked effectively even after it was coated on filter paper, to arrive at a solid-state sensor for effective solution-state detection.

### Vapor Phase Pesticide Detection

Detection of pesticides in air is relatively uncommon, due to the low vapor pressures of these pesticides and their resulting low concentrations. Nonetheless, notable fluorescence-based vapor-phase pesticide detection methods have been reported. In one example, workers who were charged with spraying of the pesticide paraquat were monitored for airborne paraquat exposure using HPLC separation with fluorescence detection (Konthonbut et al., 2020), whereas in another example, wine growers were assessed for aerosolized methyl parathion exposure using similar analytical techniques (Muttray et al., 2006).

Fluorescence-based detection was also used to determine how well certain fabrics protected against penetration by aerosolized pesticides, by combining the aerosolized pesticide with a fluorescent tracer (7-diethyl-amino-coumarin) and using solid-state fluorescence spectroscopy to track the penetration (Saleh et al., 1998). Finally, a variety of other fluorescence-based methods for pesticide detection in aerosols and airborne particles have been reported (Jongen et al., 1991; Li et al., 2004; Sivaprakasam et al., 2004).

## Conclusion and Future Outlook

Significant research efforts have demonstrated that pesticide detection can be accomplished using a variety of techniques, including fluorescence-based detection, and that such detection can be accomplished with sensitivity, selectivity, and broad-based applicability in a variety of complex environments. Despite these high levels of success, however, unsolved challenges remain, including the fact that the vast majority of fluorescence-based pesticide sensors respond to the presence of the analyte with a decrease in the observed fluorescence emission. Although such pesticide-induced fluorescence quenching enables high performance sensors, there are situations in which turn-on fluorescence sensors for pesticides might be more beneficial. Efforts to address this issue are ongoing, and include the use of pesticide-induced disruption of electron transfer that restores the electron donor’s innate luminescence (Hou et al., 2020). More broadly, efforts to solve unsolved challenges in fluorescence-based pesticide detection are expected to lead to future developments and significant advances in pesticide research.

One example of an unsolved challenge includes the fact that nearly all fluorescence-based pesticide detection methods reported to date are effective in detecting a single pesticide analyte. While there are isolated cases in which only one pesticide is present, many more situations arise in which there are mixtures of pesticides or mixtures of pesticides combined with their degradation products (Van Metre et al., 2017). Detecting multiple pesticide-based analytes simultaneously and accurately remains a challenge, especially if the goal is to detect those pesticides without requiring chromatography-based separation. To address this challenge, one may look at successes with statistical-based analysis (linear discriminant analysis (Goschnick et al., 2007) and/or principal component analysis) (Wijayanti et al., 2019), and how researchers have used such tools to detect analyte mixtures for other applications. Alternatively, using additional methods to achieve separation prior to fluorescence-based analysis may also be possible, particularly if such methods can be made to operate effectively for on-site detection scenarios (Vonderheide et al., 2009).

Additional challenges related to the use of fluorescence-based pesticide detection is that on-site fluorescence spectroscopy remains challenging. While researchers have demonstrated recent success in using portable fluorescence spectrometers (Kamel et al., 2020) and/or smartphone-based fluorimeters (Chu et al., 2020), such devices generally suffer from reduced sensitivity. As such, even with effective fluorescence-based detection, researchers often have to take samples back to the laboratory for analysis. The ability to do on-site fluorescence detection without compromising system sensitivity would provide significant advantages, and as yet remains an unsolved challenge. Currently used methods to address this issue include collecting samples on site and bringing them back to the laboratory for detailed analysis, but such methods are ineffective for unstable pesticides and for situations in which real-time information about the presence of pesticides is required.

Finally, the effective use of remote-operated sensors would also address significant challenges. In many cases, pesticide detection with remote sensors could provide critical ecosystem information. While remotely operated sensors are currently in use (Abd El-Ghany et al., 2020), they generally do not use fluorescence-based methods (Kirk and Andreescu, 2019), and so the ability to combine the advantages of fluorescence sensing with remotely operated sensors remains out of reach. Overall, the interested reader is advised to consider these challenges in designing new research projects, with the goal of solving such challenges and improving broad-based pesticide detection efforts.
